# eGDR and gastrointestinal cancer mortality in the UK Biobank: a prospective cohort study

**DOI:** 10.1186/s12885-026-16931-1

**Published:** 2026-09-05

**Authors:** Chuang Yang, Patrick S. Plum, Thomas Ebert, Jeanette Köppe, Ines Gockel, René Thieme

**Affiliations:** 1 Department of Visceral, Transplant, Thoracic and Vascular Surgery, University Hospital Leipzig, Liebigstr. 19, Leipzig, D-04103 Germany; 2 Department of Thyroid Surgery, The First Affiliated Hospital of Anhui Medical University, Hefei, Anhui 230001 China; 3 Department of General, Visceral and Transplant Surgery, University Hospital Tübingen, Tübingen, Germany; 4 Medical Department III - Endocrinology, Nephrology, Rheumatology, University of Leipzig Medical Center, Leipzig, Germany; 5 Department of Epidemiology and Biostatistics, Public and Global Health, Medical University Lausitz – Carl Thiem, Cottbus, Germany; 6 Department of Visceral Surgery, Clarunis – Universitäres Bauchzentrum Basel, Basel, Switzerland

**Keywords:** Insulin Sensitivity, Estimated Glucose Disposal Rate, Insulin Resistance, Gastrointestinal cancer mortality, Real-world evidence

## Abstract

**Background:**

This study examined the associations of estimated glucose disposal rate (eGDR), a surrogate of insulin sensitivity, and the insulin resistance indices TG/HDL-C and TyG with gastrointestinal (GI) cancer mortality in the UK Biobank.

**Methods:**

We included 369,447 participants without cancer at baseline and recorded 4,305 GI cancer-related deaths over a mean follow-up of 13.5 years. Fine-Gray models assessed associations of eGDR, TG/HDL-C, and TyG with GI cancer mortality.

**Results:**

Higher eGDR was associated with lower pooled GI cancer mortality (sHR, 0.67; 95% CI, 0.61–0.74; *P* < 0.001); associations with EC, ESCC, CRC, LC, and PC mortality remained significant after false discovery rate (FDR) correction. TyG, but not TG/HDL-C, remained associated with pooled GI cancer mortality, and both markers remained associated with LC mortality. Subgroup analyses were exploratory.

**Conclusions:**

Higher eGDR was associated with lower pooled and several site-specific GI cancer mortality outcomes, whereas TG/HDL-C and TyG associations were modest and site-specific. These findings do not establish clinical utility.

**Supplementary Information:**

The online version contains supplementary material available at https://doi.org/10.1186/s12885-026-16931-1.

## Introduction

Insulin sensitivity (IS) and insulin resistance (IR) are central determinants of metabolic health and are closely linked to cardiometabolic diseases (CMD). Higher IS is generally associated with lower risks of major cardiovascular events, whereas IR markedly increases CMD risk [1–3]. Mechanistically, impaired insulin-mediated glucose uptake leads to compensatory hyperinsulinemia and hyperglycemia, promoting systemic metabolic dysregulation and chronic inflammation and contributing to cardiometabolic morbidity and mortality [4].

Beyond CMD, accumulating evidence suggests that IS and IR may also influence cancer initiation and progression [5]. Insulin and insulin-like growth factor signaling through insulin receptors (INSR) and insulin-like growth factor-1 receptors (IGF-1R) activates oncogenic pathways, including PI3K–AKT–mTOR and MAPK, that support tumor proliferation, angiogenesis, survival, and resistance to apoptosis [6–9]. Hyperinsulinemia, a hallmark of IR, may further potentiate these pathways and strengthen the link between metabolic dysfunction and tumorigenesis [10].

Gastrointestinal (GI) cancers, including esophageal cancer (EC), gastric cancer (GC), colorectal cancer (CRC), liver cancer (LC), and pancreatic cancer (PC), remain among the most lethal malignancies worldwide [11]. In 2022, GI cancers accounted for more than 4.7 million new cases and approximately 3.2 million deaths, representing nearly one-third of global cancer mortality, and 5-year survival remains poor across sites [12, 13].

Given the frequent involvement of metabolic dysfunction in GI carcinogenesis, these cancers may be particularly sensitive to altered insulin signaling. Although previous studies have mainly examined IR in relation to GI cancer incidence [14–17], evidence linking IS or IR to GI cancer-specific mortality in initially cancer-free populations remains limited, despite the ready availability of metabolic surrogate markers.

The hyperinsulinemic-euglycemic clamp is the criterion standard for in vivo assessment of IS, but its complexity limits use in large studies [18, 19]. More accessible surrogate markers include estimated glucose disposal rate (eGDR) for IS [20, 21] and the triglyceride-to-high-density lipoprotein cholesterol ratio (TG/HDL-C) and triglyceride-glucose (TyG) index for IR [22, 23].

Lower eGDR has been associated with higher cardiovascular risk in T2DM [21, 24], whereas higher TG/HDL-C or TyG has been linked to increased risks of CRC, EC, and GC [15, 25, 26]. Whether these markers are associated with GI cancer-specific mortality remains unclear. We therefore examined their associations with GI cancer mortality in the UK Biobank.

## Materials and methods

### Study design and population

The UK Biobank is an ongoing prospective cohort that recruited more than 500,000 adults aged 37–73 years between 2006 and 2010 at 22 assessment centers in England, Scotland, and Wales. At baseline, participants completed electronic questionnaires and physical assessments and provided biological samples. The study design and data-collection methods have been described previously [27].

### Data collection and definitions

Baseline assessments included sociodemographic, lifestyle, medical, anthropometric, and biochemical data. Sociodemographic variables comprised age, sex, race and ethnicity, and socioeconomic deprivation measured using the Townsend Deprivation Index (TDI). Smoking was categorized as never, previous, or current, and alcohol consumption as never, special occasions only, one to three times per month, once or twice per week, three or four times per week, or daily or almost daily. Total physical activity was calculated as metabolic equivalent task minutes per week (MET-min/week) from self-reported walking and moderate- and vigorous-intensity activity. Diet quality was summarized using a validated score based on nine food groups (range, 0–9), with higher scores indicating a healthier dietary pattern [28]. These lifestyle variables were selected a priori because they may confound associations between metabolic status and GI cancer outcomes. Medical history included hypertension, cardiovascular disease (CVD), and use of antihypertensive, glucose-lowering, and lipid-lowering medications. Type 2 diabetes mellitus (T2DM) was identified from self-report, glucose-lowering medication use, or hospital records coded as ICD-10 E11. Anthropometric measurements included height, weight, body mass index (BMI), waist circumference (WC), and duplicate blood pressure measurements; mean systolic and diastolic values were used. Baseline proton pump inhibitor (PPI) use was obtained from UK Biobank Data-Field 20,003 and coded as any versus none.

Blood samples were analyzed in the UK Biobank central laboratory using standardized protocols [29]. Glucose, glycated hemoglobin (HbA1c), triglycerides, high-density lipoprotein cholesterol (HDL-C), and C-reactive protein (CRP) were used in this analysis. eGDR (mg/kg/min) was calculated as 21.158 − (0.09 × WC [cm]) − (3.407 × hypertension [yes = 1, no = 0]) − (0.551 × HbA1c [%]) [20]. TG/HDL-C was calculated as triglycerides (mg/dL) divided by HDL-C (mg/dL), and TyG as ln[triglycerides (mg/dL) × glucose (mg/dL)/2] [15]. Hypertension was defined by self-reported diagnosis, mean systolic blood pressure ≥ 140 mmHg, mean diastolic blood pressure ≥ 90 mmHg, antihypertensive medication use, or ICD-10 codes I10–I15 [30].

### Outcome assessment

The primary outcome was mortality from all GI cancers (ICD-10 C15–C25). Secondary outcomes were mortality from esophageal cancer (EC; C15), gastric cancer (GC; C16), colorectal cancer (CRC; C18–C20), liver cancer (LC; C22), and pancreatic cancer (PC; C25). Mortality from esophageal adenocarcinoma (EAC) and esophageal squamous cell carcinoma (ESCC) was also analyzed; histological classifications are provided in Table S1. Because these malignancies are biologically heterogeneous, the pooled outcome was treated as an overall summary and site-specific estimates were analyzed and interpreted separately. Participants were followed from baseline until death or December 19, 2022, whichever occurred first.

### Selection criteria

**Fig. 1:**
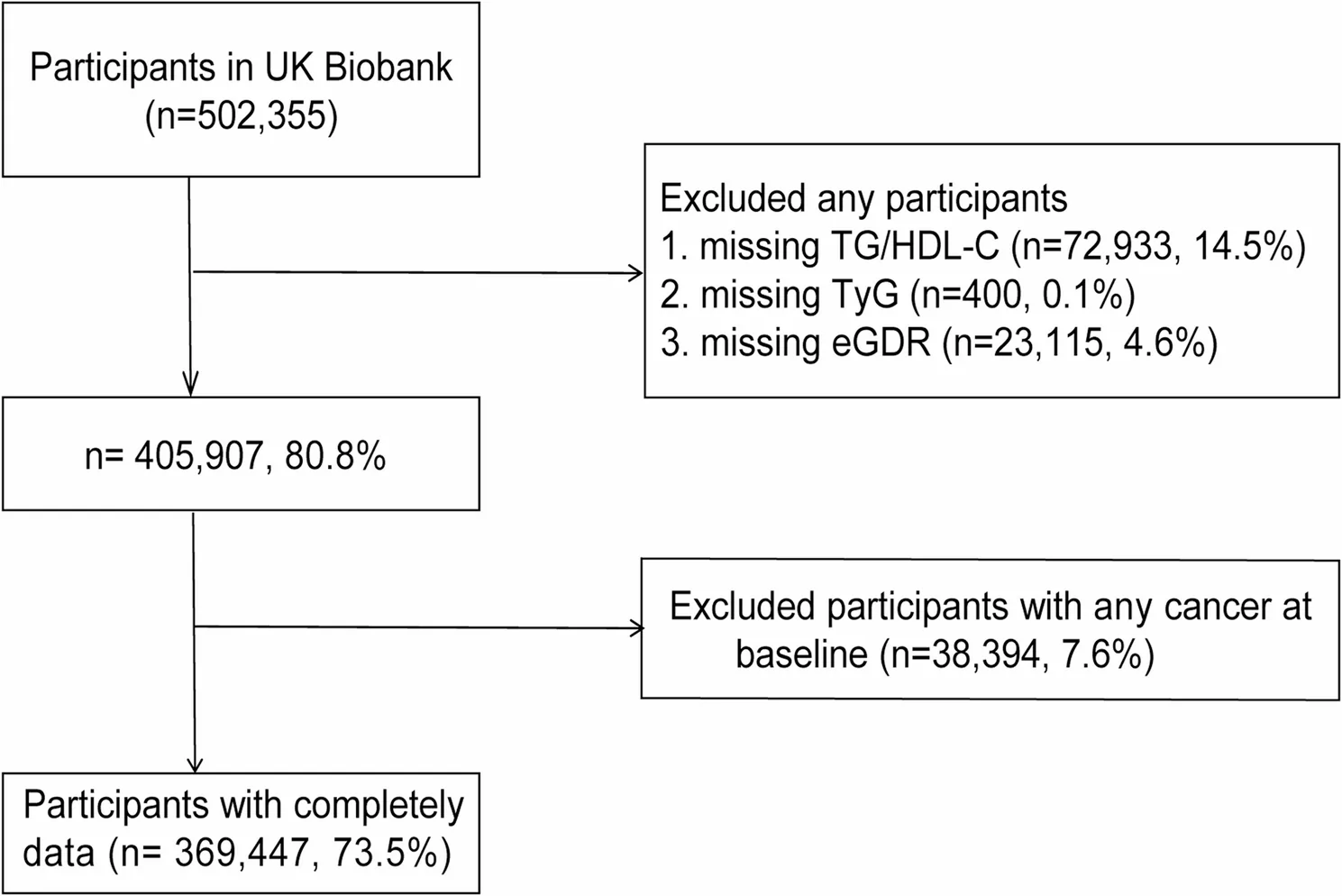
Flow diagram of participant selection

Among 502,355 participants, 96,448 lacked at least one exposure measurement (TG/HDL-C, *n* = 72,933; TyG, *n* = 400; or eGDR, *n* = 23,115), leaving 405,907 participants with complete exposure data. After excluding 36,460 participants with a cancer diagnosis before baseline, the final analytic sample comprised 369,447 participants (Fig. 1). Baseline characteristics were compared between participants with and without complete exposure data to assess potential selection bias (Table S2).

### Descriptive analysis

Baseline characteristics were compared between participants with and without complete exposure data to assess potential selection bias (Table S2). For each incomplete covariate, a binary missingness indicator was modeled using fully observed baseline characteristics. Little’s test rejected missing completely at random (MCAR; χ²=9,304, df = 40, *P* < 0.001), and missingness was associated with observed characteristics for all incomplete covariates (Table S3), supporting missing at random (MAR) as a working assumption. Missing covariates were imputed using random forest-based MICE with five imputed datasets (m = 5); predictors are listed in Table S4. Outcome indicators and follow-up time were not included in the imputation models. Continuous variables are presented as mean ± SD and categorical variables as n (%). eGDR, TG/HDL-C, and TyG were standardized to Z scores and also analyzed in quartiles.

### Primary endpoint – univariate and multivariable analyses

Deaths from causes other than the outcome under study were treated as competing events. Cumulative incidence functions across quartiles of eGDR, TG/HDL-C, and TyG were estimated using the Aalen-Johansen method and compared using Gray’s test. Fine-Gray competing risk regression estimated subdistributional hazard ratios (sHRs) and 95% confidence intervals (CIs) per 1-SD increase and across quartiles (Q1 as the reference). The sHR was interpreted as an association with the cumulative incidence of GI cancer mortality in the presence of deaths from causes other than the outcome under study. By contrast, the cause-specific Cox model used in sensitivity analysis estimated hazard ratios (HRs) for the instantaneous hazard of GI cancer mortality among participants who remained free of both GI cancer death and the competing event. Accordingly, sHR is used for Fine-Gray estimates and HR for cause-specific Cox estimates. The proportional hazards assumption for the cause-specific Cox models was assessed using scaled Schoenfeld residuals, with no material violations identified. Model 1 adjusted for age, sex, and ethnicity. Model 2 additionally adjusted for BMI, TDI, total MET-min/week, smoking status, alcohol consumption frequency, antihypertensive, lipid-lowering, and glucose-lowering medication use, T2DM, hypertension, CVD, family history of cancer, and diet score. Covariates were selected a priori according to their established associations with metabolic status and GI cancer outcomes and with reference to previous population-based studies [15, 25]. Model 2 was retained as a conservative, extensively adjusted model; potential overlap between its covariates and the metabolic indices was evaluated in reduced-covariate sensitivity analyses.

### Sensitivity and subgroup analyses for primary endpoints

A series of sensitivity analyses were conducted to assess the robustness of the findings. First, participants who died from GI cancer within the first two years of follow-up were excluded to address potential reverse causation. Second, analyses were repeated using complete cases only. Third, multiple imputation by chained equations with predictive mean matching (MICE-PMM) was applied to account for missing data. Fourth, models were further adjusted for baseline PPI use. Fifth, cause-specific Cox models were fitted with deaths from causes other than the outcome under study censored, as an alternative to the Fine–Gray approach. Sixth, eGDR analyses were repeated after excluding participants with T2DM.

In addition, to mitigate potential multicollinearity and overadjustment due to overlap between metabolic indices and cardiometabolic covariates, two further sensitivity analyses with reduced covariate sets were performed. The first excluded BMI, type 2 diabetes, hypertension, CVD, and related medication use from the model; the second excluded smoking status, alcohol consumption frequency, MET score, and dietary score to evaluate whether effect estimates were sensitive to lifestyle adjustment.

Subgroup analyses assessed potential effect modification by sex, age (< 60 or ≥ 60 years), BMI (< 30 or ≥ 30 kg/m²), TDI ( < − 1.31 or ≥ − 1.31), CRP (< 2.57 or ≥ 2.57 mg/L), smoking status (never vs. previous/current), alcohol consumption (never vs. any), prediabetes, and lipid-lowering medication use. The age and BMI cutoffs were prespecified as clinically interpretable thresholds, whereas TDI and CRP were dichotomized at their cohort medians to avoid outcome-driven cut point selection and obtain comparably sized subgroups. Prediabetes was defined according to published criteria [31]. Fully adjusted Fine-Gray models were fitted within each subgroup, and interaction P values were obtained using one-degree-of-freedom Wald tests. Statistical significance in one stratum but not another was not considered evidence of effect modification without support from the corresponding interaction test. Given the number of comparisons, subgroup and interaction analyses were considered exploratory.

### Additional analyses

Restricted cubic spline (RCS) terms with knots at the 10th, 50th, and 90th percentiles were applied to assess departures from linearity in the associations between each continuous marker and GI cancer mortality. The spline analyses were considered exploratory, and the curve shapes were interpreted cautiously, particularly at the extremes of the exposure distributions where uncertainty was greater. Incident GI cancers were ascertained using the same site definitions and esophageal histological classification as the mortality outcomes. Death before diagnosis was treated as a competing event, and fully adjusted Fine–Gray models examined associations of a 1-SD increase in eGDR with incident GI cancer.

All analyses were performed using R version 4.3.1. Multiple comparisons were adjusted for using the Benjamini-Hochberg procedure to control the false discovery rate (FDR) across 24 fully adjusted prespecified exposure-mortality comparisons (three metabolic markers × eight GI cancer mortality outcomes), with an FDR-adjusted P value (P_adj) < 0.05 defining statistical significance. Correction for the secondary eGDR incidence analysis was applied separately across the eight outcomes. Subgroup and interaction analyses were considered exploratory and were not corrected for multiple comparisons.

## Results

### Characteristics of the study cohort

A total of 369,447 participants without cancer at baseline were included. Their mean age was 56.3 ± 8.1 years, 47.3% were male, and 94.8% were White (Table 1). During a mean follow-up of 13.5 years, 4,305 GI cancer-related deaths occurred, including 704 from EC, 366 from GC, 1,361 from CRC, 537 from LC, and 1,146 from PC. Compared with participants who remained alive or died from non-GI causes, those who died from GI cancer were older and more likely to be male and had higher BMI and greater prevalences of T2DM, hypertension, and CVD. They also had lower eGDR and higher TG/HDL-C and TyG values at baseline.

**Table 1:** Baseline demographic and clinical characteristics

Characteristics	Total (*n* = 369,447)	Alive or died from non-GI causes (*n* = 365,142)	GI cancer deaths (*n* = 4,305)
Age, years	56.3 ± 8.1	56.2 ± 8.1	61.1 ± 6.4
Male, N (%)	174,585 (47.3%)	171,977 (47.1%)	2,608 (60.6%)
White, N (%)	350,121 (94.8%)	345,955 (94.8%)	4,166 (96.8%)
Total MET-min/week	2642.4 ± 2653.6	2642.9 ± 2653.8	2600.0 ± 2638.6
Townsend deprivation index	-1.3 ± 3.1	-1.3 ± 3.1	-1.2 ± 3.2
Body mass index (kg/m^2^)	27.5 ± 4.8	27.4 ± 4.8	28.5 ± 5.2
Diet score	5.1 ± 1.6	5.1 ± 1.6	5.2 ± 1.6
Diabetes mellitus, N (%)	22,339 (6.1%)	21,784 (6.0%)	555 (12.9%)
Hypertension, N (%)	198,248 (53.7%)	195,329 (53.5%)	2,919 (67.8%)
Cardiovascular disease, N (%)	28,449 (7.7%)	27,912 (7.6%)	537 (12.5%)
Lipid-lowering medication use, N (%)	63,714 (17.3%)	62,555 (17.1%)	1,159 (26.9%)
Antihypertensive medication use, N (%)	75,674 (20.5%)	74,301 (20.4%)	1,373 (31.9%)
Antidiabetic medication use, N (%)	13,533 (3.7%)	13,186 (3.6%)	347 (8.1%)
Smoking status, N (%)			
Never	148,916 (40.3%)	147,535 (40.4%)	1,381 (32.1%)
Previous	181,469 (49.1%)	179,183 (49.1%)	2,286 (53.1%)
Current	39,062 (10.6%)	38,424 (10.5%)	638 (14.8%)
Alcohol intake frequency, N (%)			
Never	29,333 (7.9%)	28,923 (7.9%)	410 (9.5%)
Special occasions only	41,740 (11.3%)	41,219 (11.3%)	521 (12.1%)
One to three times a month	41,183 (11.2%)	40,779 (11.2%)	404 (9.4%)
Once or twice a week	95,749 (25.9%)	94,761 (25.9%)	988 (22.9%)
Three or four times a week	85,999 (23.3%)	85,070 (23.3%)	929 (21.6%)
Daily or almost daily	75,443 (20.4%)	74,390 (20.4%)	1,053 (24.5%)
Family history of cancer, N (%)	110,721 (30.0%)	109,356 (29.9%)	1,365 (31.7%)
TG/HDL-C	3.2 ± 2.6	3.2 ± 2.6	3.7 ± 3.0
TyG index	8.7 ± 0.6	8.7 ± 0.6	8.9 ± 0.6
eGDR, mg/kg/min	8.2 ± 2.5	8.2 ± 2.5	7.1 ± 2.5

*GI cancer* gastrointestinal cancer, *TG/HDL-C* triglycerides/high-density lipoprotein cholesterol ratio, *TyG* triglyceride-glucose, *eGDR* estimated glucose disposal rate

### Association of eGDR, TG/HDL-C and TyG with GI cancer mortality

**Fig. 2:**
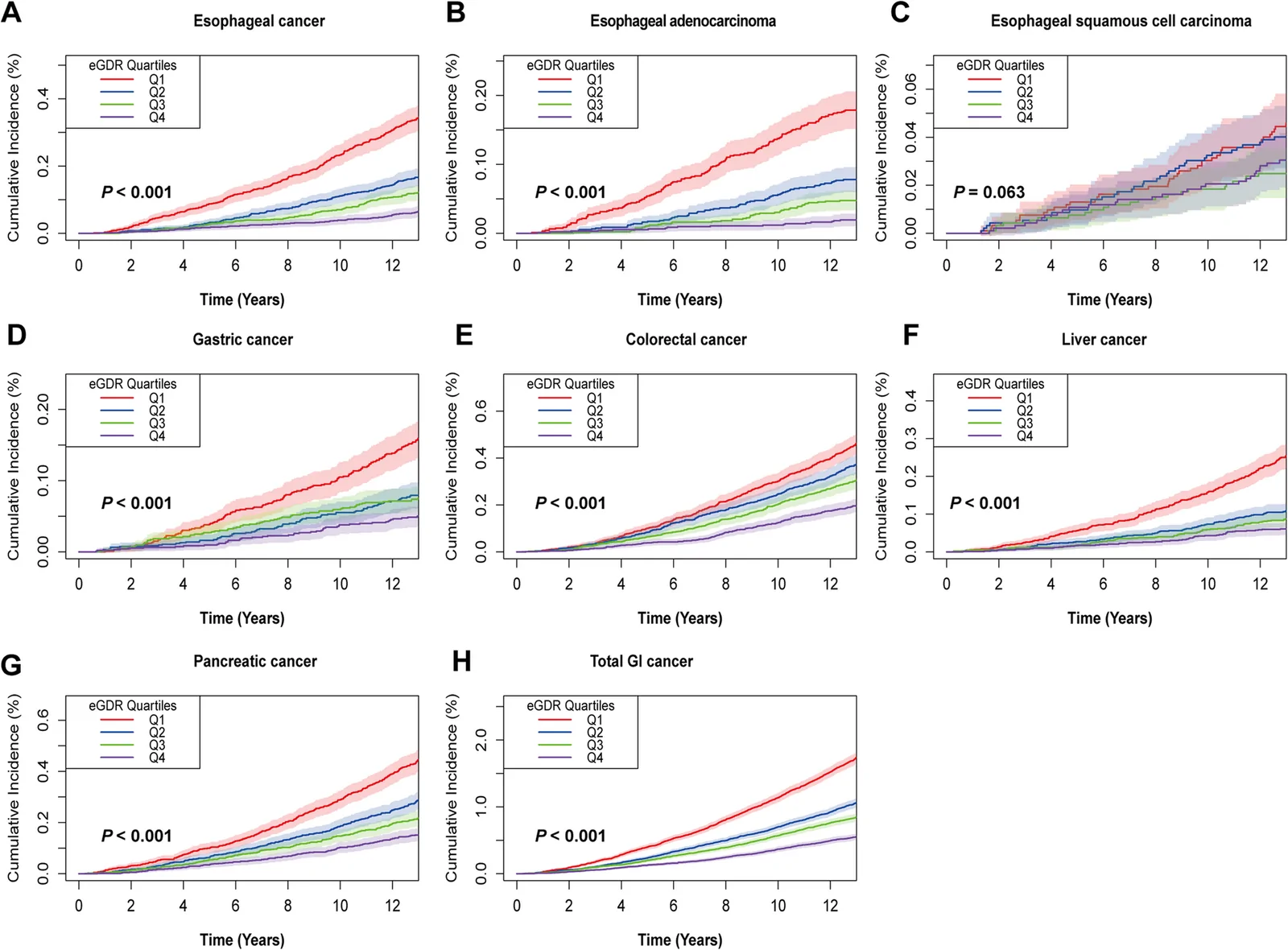
Cumulative incidence curves of gastrointestinal cancer mortality by eGDR quartiles. **A** Esophageal cancer, (**B**) Esophageal adenocarcinoma, (**C**) Esophageal squamous cell carcinoma, (**D**) Gastric cancer, (**E**) Colorectal cancer, (**F**) Liver cancer, (**G**) Pancreatic cancer, and (**H**) Pooled GI cancer mortality. Only the cumulative incidence of the outcome of interest is shown; competing deaths were incorporated in the estimation but are not displayed

The 10-year cumulative incidence of pooled GI cancer mortality was 0.69% (95% CI, 0.67%–0.72%): 0.11% (95% CI, 0.10%–0.13%) for EC, 0.06% (95% CI, 0.05%–0.08%) for GC, 0.22% (95% CI, 0.20%–0.24%) for CRC, 0.08% (95% CI, 0.07%–0.10%) for LC, and 0.18% (95% CI, 0.17%–0.19%) for PC. Cumulative incidence decreased across increasing eGDR quartiles (Fig. 2) and increased across TG/HDL-C and TyG quartiles (Figs. S1 and S2).

After full adjustment in Model 2, each 1-SD increase in eGDR was associated with a lower cumulative incidence of pooled GI cancer mortality (sHR, 0.67; 95% CI, 0.61–0.74; *P* < 0.001). This association and those with EC, ESCC, CRC, LC, and PC mortality remained significant after FDR correction (all P_adj < 0.05; Table 2); the nominal association with GC mortality (P_adj > 0.05) did not. TyG remained significant with pooled GI cancer mortality after FDR correction (sHR, 1.05; 95% CI, 1.01–1.08; P_adj < 0.05), whereas TG/HDL-C did not (sHR, 1.03; 95% CI, 1.00–1.07; P_adj > 0.05). Both TG/HDL-C and TyG remained significant with LC mortality after FDR correction; neither marker remained associated with the other site-specific outcomes.

**Table 2:** Associations of per-SD increases in eGDR, TG/HDL-C, and TyG with GI cancer mortality estimated using Fine-Gray competing risk models

Types	eGDR				TG/HDL-C				TyG			
	Model 1		Model 2		Model 1		Model 2		Model 1		Model 2	
	sHR (95% CI)	*P*	sHR (95% CI)	*P*	sHR (95% CI)	*P*	sHR (95% CI)	*P*	sHR (95% CI)	*P*	sHR (95% CI)	*P*
EC	0.72 (0.66–0.79)	< 0.001	0.66 (0.55–0.80)	< 0.001*	1.06 (1.00-1.13)	0.048	0.97 (0.91–1.04)	0.44	1.13 (1.05–1.22)	< 0.001	1.02 (0.95–1.10)	0.59
EAC	0.63 (0.55–0.72)	< 0.001	0.75 (0.51–1.11)	0.15	1.12 (1.04–1.20)	0.001	1.00 (0.92–1.09)	0.97	1.27 (1.14–1.41)	< 0.001	1.09 (0.98–1.22)	0.11
ESCC	0.96 (0.78–1.19)	0.71	0.64 (0.47–0.86)	0.003*	0.85 (0.63–1.13)	0.268	0.90 (0.67–1.21)	0.48	0.82 (0.66–1.01)	0.057	0.85 (0.67–1.07)	0.17
GC	0.85 (0.75–0.96)	0.009	0.71 (0.51–0.99)	0.043	1.11 (1.02–1.20)	0.014	1.03 (0.93–1.13)	0.57	1.09 (0.98–1.22)	0.11	1.00 (0.89–1.12)	0.97
CRC	0.87 (0.82–0.93)	< 0.001	0.67 (0.57–0.78)	< 0.001*	1.07 (1.02–1.12)	0.007	1.04 (0.99–1.10)	0.16	1.08 (1.02–1.14)	0.007	1.03 (0.97–1.10)	0.31
LC	0.63 (0.56–0.71)	< 0.001	0.65 (0.54–0.79)	< 0.001*	1.25 (1.18–1.32)	< 0.001	1.12 (1.04–1.20)	0.002*	1.41 (1.29–1.55)	< 0.001	1.14 (1.04–1.25)	0.006*
PC	0.80 (0.75–0.86)	< 0.001	0.68 (0.58–0.79)	< 0.001*	1.09 (1.03–1.14)	0.002	1.02 (0.96–1.08)	0.61	1.15 (1.08–1.20)	< 0.001	1.05 (0.99–1.12)	0.13
GI	0.79 (0.76–0.82)	< 0.001	0.67 (0.61–0.74)	< 0.001*	1.11 (1.08–1.13)	< 0.001	1.03 (1.00-1.07)	0.026	1.15 (1.12–1.19)	< 0.001	1.05 (1.01–1.08)	0.005*

*eGDR* estimated glucose disposal rate, *TG/HDL-C* triglycerides/high-density lipoprotein cholesterol ratio, *TyG* triglyceride-glucose index, *sHR* subdistribution hazard ratio, *GI* gastrointestinal, *EC* esophageal cancer, *EAC* esophageal adenocarcinoma, *ESCC* esophageal squamous cell carcinoma, *GC* gastric cancer, *CRC* colorectal cancer, *LC* liver cancer, *PC* pancreatic cancer, *SD* standard deviation

Model 1 adjusted for age, sex, and race and ethnicity. Model 2 additionally adjusted for BMI, Townsend Deprivation Index, total MET-min/week, smoking status, alcohol consumption frequency, antihypertensive, lipid-lowering, and glucose-lowering medication use, T2DM, hypertension, CVD, family history of cancer, and diet score. *Nominal Model 2 P values that remained significant after Benjamini–Hochberg FDR correction across 24 comparisons (P_adj < 0.05)

### Sensitivity analysis

The principal findings were broadly consistent after excluding deaths within the first two years (Table S5), restricting analyses to complete cases (Table S6), using MICE-PMM (Table S7), additionally adjusting for PPI use (Table S8), and fitting cause-specific Cox models (Table S9). Higher eGDR also remained inversely associated with pooled GI cancer mortality in the strictly non-diabetic subset (Table S10). Results were broadly similar after excluding potentially overlapping cardiometabolic covariates (Table S11) or lifestyle covariates (Table S12), indicating that the main patterns were not materially dependent on a single covariate set.

### Subgroup analyses

Subgroup-specific estimates and formal interaction tests are presented in Tables S13–S21. Across most strata, the directions of the associations were broadly consistent with the primary findings. Although several interaction *P* values were nominally significant, no consistent pattern of effect modification emerged across the metabolic markers, cancer outcomes, or stratification factors. Given the large number of tests and the absence of correction for multiple comparisons, these subgroup analyses were considered exploratory and should be interpreted cautiously.

### Restricted cubic spline analyses

**Fig. 3:**
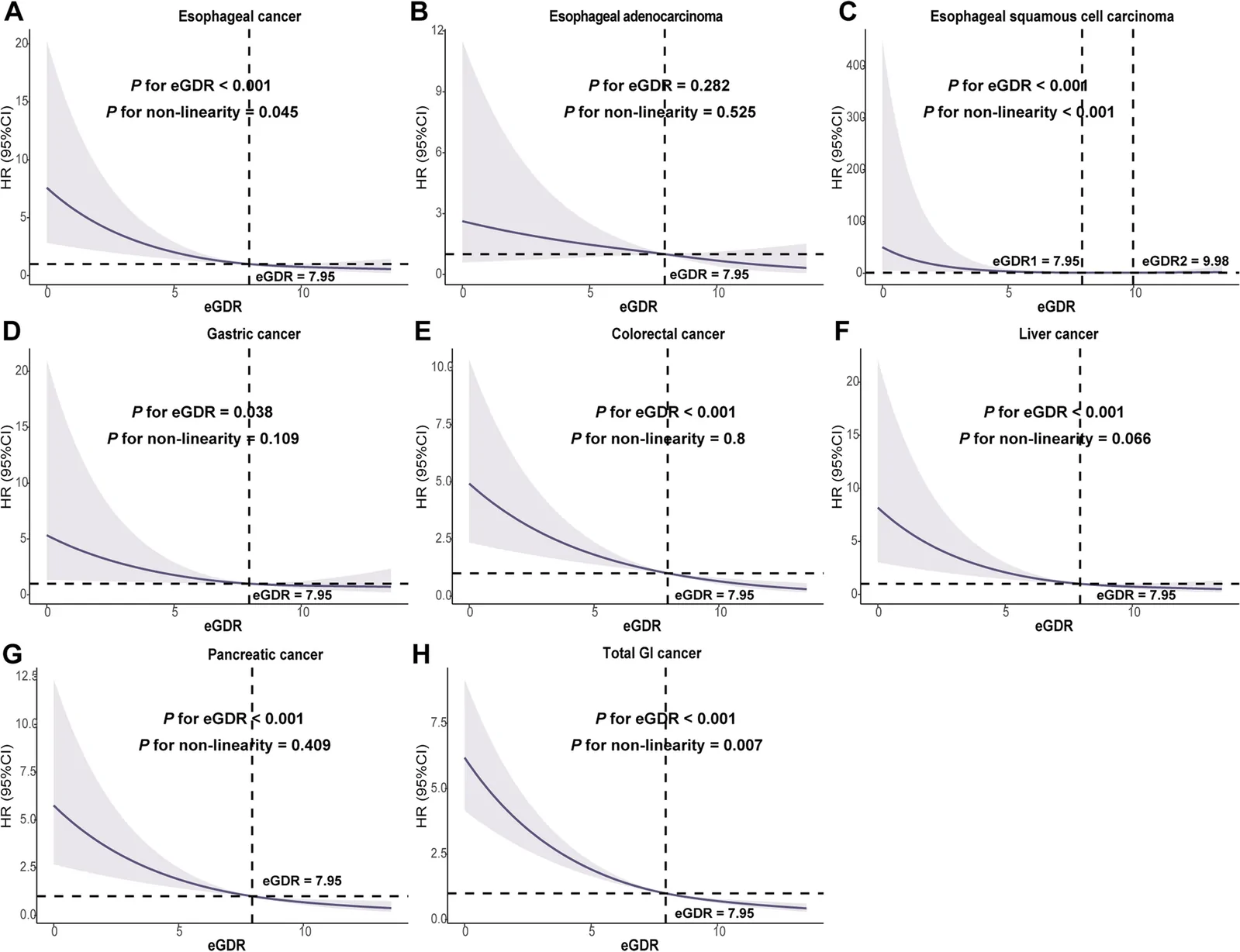
Association of eGDR with gastrointestinal cancer mortality using restricted cubic splines. **A** Esophageal cancer, (**B**) Esophageal adenocarcinoma, (**C**) Esophageal squamous cell carcinoma, (**D**) Gastric cancer, (**E**) Colorectal cancer, (**F**) Liver cancer, (**G**) Pancreatic cancer, and (**H**) Pooled GI cancer mortality. Curves show fully adjusted cause-specific HRs (solid lines) with 95% CIs (shaded areas); knots were placed at the 10th, 50th, and 90th percentiles

After full adjustment, RCS analyses indicated nonlinearity for eGDR in relation to EC, ESCC, and pooled GI cancer mortality (*P* for nonlinearity = 0.045, < 0.001, and 0.007, respectively; Fig. 3). For EC and pooled GI cancer mortality, the associations remained monotonic inverse, with steeper declines at lower eGDR levels and flatter slopes at higher levels, rather than U-shaped or J-shaped patterns. TG/HDL-C showed nominal nonlinearity for EAC and ESCC, but not for LC or pooled GI cancer mortality (Fig. S3). TyG showed nonlinearity for CRC, LC, and pooled GI cancer mortality (Fig. S4). Given the greater uncertainty at the exposure extremes, the spline findings were considered exploratory and were not interpreted as clinically meaningful thresholds or turning points. In the secondary incidence analysis, eGDR was inversely associated with incident pooled GI cancer and EC, EAC, CRC, LC, and PC after separate FDR correction (Table S22).

## Discussion

To our knowledge, this is the first large prospective cohort study to systematically examine the associations of eGDR, TG/HDL-C, and TyG with GI cancer mortality. Higher eGDR was associated with lower pooled GI cancer mortality and with mortality from several individual cancer sites, including EC, ESCC, CRC, LC, and PC, after FDR correction. However, the associations were not observed consistently across all sites, particularly for EAC and GC. The pooled estimate should therefore be interpreted as an overall summary of GI cancer mortality rather than as evidence of a common association across biologically distinct malignancies.

The associations of TG/HDL-C and TyG were weaker and more restricted than those observed for eGDR. After FDR correction, TyG remained associated with pooled GI cancer mortality, whereas TG/HDL-C did not, and both markers remained associated with LC mortality. Associations with the other site-specific outcomes were largely attenuated after full adjustment and correction for multiple comparisons. Taken together, these findings suggest that eGDR showed a broader association with GI cancer mortality, whereas the associations of TG/HDL-C and TyG were modest and largely site-specific. The principal patterns were broadly consistent across sensitivity analyses, although the observational design and modest effect sizes, particularly for TG/HDL-C and TyG, preclude conclusions regarding clinical utility.

Among the exploratory subgroup analyses, age showed the most consistent nominal interaction pattern for eGDR, with stronger inverse associations among participants younger than 60 years; however, these findings were not corrected for multiple comparisons and require independent confirmation. Besides, although some associations showed statistical evidence of nonlinearity, the RCS analyses did not support clear clinically meaningful thresholds and were therefore interpreted as exploratory.

Previous studies of individual GI cancers have linked HOMA-IR with pancreatic cancer mortality and with primary liver cancer or chronic liver disease mortality [32, 33]. Research on eGDR has largely focused on all-cause and cardiovascular mortality, generally reporting inverse associations [34, 35], and one study suggested stronger mortality prediction by eGDR than by TG/HDL-C or TyG [36]. The present findings extend this epidemiological evidence to GI cancer mortality. However, this study evaluated associations rather than prediction; the reproducibility, calibration, discrimination, and incremental prognostic value of eGDR were not assessed.

TG/HDL-C and TyG reflect an adverse lipid–glucose milieu and are widely used as IR surrogates. Elevated triglycerides may impair skeletal-muscle glucose uptake and contribute to systemic metabolic stress [37]. Higher TG/HDL-C has been associated with shorter survival in non-small-cell lung cancer [38], and both markers have been linked to all-cause, cardiovascular, and COVID-19-related mortality in non-cancer populations [39–42]. The modest associations observed in the present study extend this literature to GI cancer mortality but do not establish clinical importance or predictive utility.

Potential mechanisms are multifactorial but were not directly tested. Hyperinsulinemia may activate INSR-A and IGF-1R signaling, promoting proliferation, survival, invasion, and resistance to apoptosis [7, 43–45]. Downstream dysregulation of PI3K–AKT–mTOR and MAPK pathways may contribute to aggressive tumor behavior [46, 47]. Chronic inflammation, altered glucose and lipid metabolism, oxidative stress, and immune dysregulation are additional plausible pathways; higher eGDR has been associated with a more favorable metabolic and inflammatory profile [48]. Recent prospective studies have examined endogenous redox-related biomarkers, including bilirubin, albumin, uric acid, and creatinine, in relation to cancer risk [49, 50]. The JPHC Study reported inverse associations of albumin and bilirubin with total cancer risk in selected analyses but no dose-responsive association for uric acid [50], whereas a Korean cohort found nonlinear, age- and cancer-site-dependent patterns across these four biomarkers [49]. Additionally, a meta-analysis also linked higher serum uric acid and gout to increased colorectal cancer incidence [51], underscoring that these biomarkers may reflect antioxidant activity as well as metabolic, hepatic, renal, and inflammatory states and cannot be interpreted as direct measures of oxidative stress. These mechanisms should be regarded as hypotheses rather than explanations established by the present data.

eGDR, TG/HDL-C, and TyG can be derived from routinely collected measurements, which makes them useful for population-level research on metabolic correlates of GI cancer mortality. Nevertheless, eGDR is a surrogate rather than a criterion-standard measure of insulin sensitivity, and the present associations do not demonstrate individual-level prediction or support clinical risk stratification. Dedicated external validation and prediction studies are required before clinical application.

Several limitations warrant consideration. First, eGDR, TG/HDL-C, and TyG were measured only at baseline, so changes before and after cancer diagnosis were not captured. Second, cancer stage and treatment were unavailable. Third, the UK Biobank includes predominantly White participants and is affected by healthy-volunteer selection, limiting generalizability. Fourth, the observational design precludes causal inference, and residual confounding remains possible. Fifth, site-specific cancer factors, including *Helicobacter pylori* infection, viral hepatitis, cirrhosis, pancreatitis, colorectal cancer screening history, and aspirin use, were not included in the common covariate set because their relevance varies across cancer sites. In addition, cirrhosis and pancreatitis may lie on the causal pathway, whereas aspirin use is susceptible to confounding by indication. Residual confounding by these factors therefore cannot be excluded. Sixth, the pooled outcome combines biologically distinct cancers, although site-specific analyses were performed. Seventh, the missing-at-random assumption cannot be verified and missing-not-at-random mechanisms cannot be excluded, despite complete-case and alternative-imputation analyses. Eighth, TG/HDL-C and TyG may be affected by lipid- and glucose-lowering therapy. Finally, direct measures of oxidative stress, total antioxidant capacity, and redox balance were unavailable; bilirubin and uric acid are indirect, nonspecific markers and were not added as mechanistic measures.

## Conclusions

In this large prospective cohort study, higher eGDR was associated with lower pooled GI cancer mortality and mortality from several individual sites, whereas associations of TG/HDL-C and TyG were modest and largely limited to LC. These findings describe population-level epidemiological associations rather than established clinical utility. Further studies should evaluate reproducibility, mechanisms, and incremental predictive value before these markers are considered for clinical risk stratification.

## Supplementary Information

Supplementary Material 1.

## Data Availability

Data from the UK Biobank (https://www.ukbiobank.ac.uk) are available to all researchers upon making an application. This study utilized the UK Biobank resource (application number: 107335).

## Abbreviations

IS: Insulin sensitivity
IR: Insulin resistance
eGDR: Estimated glucose disposal rate
TG/HDL-C: Triglycerides-to-high-density lipoprotein cholesterol ratio
TyG: Triglyceride-glucose index
T2DM: Type 2 diabetes mellitus
CVD: Cardiovascular disease
CI: Confidence interval
HR: Cause-specific hazard ratio
sHR: Subdistribution hazard ratio
SD: Standard deviation
GI: Gastrointestinal
EC: Esophageal cancer
GC: Gastric cancer
CRC: Colorectal cancer
LC: Liver cancer
PC: Pancreatic cancer
UKB: UK Biobank
BMI: Body mass index
TDI: Townsend deprivation index
ICD-10: International Classification of Diseases, 10th Revision
