# Supplementary material for: eGDR and gastrointestinal cancer mortality in the UK Biobank: a prospective cohort study
Source: BMC Cancer. 2026 Sep 5;26:1060. doi: 10.1186/s12885-026-16931-1 (PMC13545786; doi:10.1186/s12885-026-16931-1)
Supplement: Supplementary file 1 [file 12885_2026_16931_MOESM1_ESM.docx]

**eGDR and gastrointestinal cancer mortality in the UK Biobank: a prospective cohort study**

[Table S1. ICD-10 code and histological subtypes of esophageal cancer in UK Biobank.	3](#_Toc234921997)

[Table S2. Baseline demographic and clinical characteristics between participants with and without exposure data.	4](#_Toc234921998)

Table S3. Information on missing covariates among 369,447 UK Biobank participants.	6

[Table S4. Variables included in the multiple imputation model.	7](#_Toc234921999)

Table S5. Sensitivity analysis of associations of per-SD increases in eGDR, TG/HDL-C, and TyG with GI cancer mortality after excluding participants who died from GI cancer within the first 2 years.	9

Table S6. Sensitivity analysis of associations of per-SD increases in eGDR, TG/HDL-C, and TyG with GI cancer mortality in the complete-case analysis.	10

Table S7. Sensitivity analysis of associations of per-SD increases in eGDR, TG/HDL-C, and TyG with GI cancer mortality using multiple imputation by chained equations with predictive mean matching (MICE-PMM).	11

Table S8. Sensitivity analysis of associations of per-SD increases in eGDR, TG/HDL-C, and TyG with GI cancer mortality with additional adjustment for baseline proton pump inhibitor use.	12

Table S9. Sensitivity analysis of associations of per-SD increases in eGDR, TG/HDL-C, and TyG with GI cancer mortality using cause-specific Cox proportional hazards models.	13

Table S10. Sensitivity analysis of the association of a per-SD increase in eGDR with GI cancer mortality after excluding participants with T2DM.	14

[Table S11. Sensitivity analysis of the associations of per-SD increases in eGDR, TG/HDL-C, and TyG with gastrointestinal cancer mortality after excluding potentially overlapping metabolic covariates.	15](#_Toc234922006)

[Table S12. Sensitivity analysis of the associations of per-SD increases in eGDR, TG/HDL-C, and TyG with gastrointestinal cancer mortality after excluding lifestyle covariates.	16](#_Toc234922007)

Table S13. Associations of per-SD increases in eGDR, TG/HDL-C, and TyG with GI cancer mortality stratified by sex.	17

Table S14. Associations of per-SD increases in eGDR, TG/HDL-C, and TyG with GI cancer mortality stratified by age.	18

Table S15. Associations of per-SD increases in eGDR, TG/HDL-C, and TyG with GI cancer mortality stratified by BMI.	19

Table S16. Associations of per-SD increases in eGDR, TG/HDL-C, and TyG with GI cancer mortality stratified by TDI.	20

Table S17. Associations of per-SD increases in eGDR, TG/HDL-C, and TyG with GI cancer mortality stratified by CRP.	21

Table S18. Associations of per-SD increases in eGDR, TG/HDL-C, and TyG with GI cancer mortality stratified by smoking status.	22

Table S19. Associations of per-SD increases in eGDR, TG/HDL-C, and TyG with GI cancer mortality stratified by alcohol consumption.	23

Table S20. Associations of per-SD increases in eGDR, TG/HDL-C, and TyG with GI cancer mortality stratified by prediabetes status.	24

Table S21. Associations of per-SD increases in eGDR, TG/HDL-C, and TyG with GI cancer mortality stratified by lipid-lowering medication use.	25

Table S22. Association of a per-SD increase in eGDR with incident GI cancer estimated using Fine–Gray competing-risk models.	26

[Figure S1. Cumulative incidence curves of gastrointestinal (GI) cancer mortality by TG/HDL-C quartiles.	27](#_Toc234922018)

[Figure S2. Cumulative incidence curves of gastrointestinal (GI) cancer mortality by TyG quartiles.	28](#_Toc234922019)

Figure S3. Association of TG/HDL-C with gastrointestinal (GI) cancer mortality using restricted cubic splines.	29

Figure S4. Association of TyG with gastrointestinal (GI) cancer mortality using restricted cubic splines.	30

| Table S1. ICD-10 code and histological subtypes of esophageal cancer in UK Biobank. | | | |
| --- | --- | --- | --- |
| **Cancer Type** | **ICD-10 code** | **Histological subtype** | **UK Biobank data field 40011 values** |
| Esophageal cancer | C15.0-15.9 | Adenocarcinoma (EAC) | 8140, 8144, 8210, 8260, 8323, 8480, 8211, 8574, 8481, 8145, 8490 |
|  |  | Squamous cell carcinoma (ESCC) | 8070, 8071, 8076 |
|  |  | Other types | 8000, 8010, 8020, 8246, 8560, 8012, 8041, 8720, 8990, 8045 |

| Table S2. Baseline demographic and clinical characteristics between participants with and without exposure data. | | | |
| --- | --- | --- | --- |
| **Characteristics** | **Non-missing (n=405,907)** | **Missing (n=96,448)** | ***P*** |
| Age, years | 56.56 ± 8.09 | 56.42 ± 8.12 | <0.001 |
| Total MET-min/week | 2647.39 ± 2654.77 | 2632.19 ± 2644.53 | 0.168 |
| Townsend deprivation index | -1.33 ± 3.07 | -1.16 ± 3.18 | <0.001 |
| Body mass index (kg/m²) | 27.43 ± 4.78 | 27.45 ± 4.90 | 0.193 |
| Diet score | 5.06 ± 1.55 | 5.04 ± 1.54 | <0.001 |
| Sex, N (%) |  |  | <0.001 |
| Women | 218,351 (53.79%) | 54,940 (56.96%) |  |
| Men | 187,556 (46.21%) | 41,508 (43.04%) |  |
| Race, N (%) |  |  | <0.001 |
| Non-white | 20,012 (4.9%) | 7,005 (7.3%) |  |
| White | 384,027 (94.6%) | 88,534 (91.8%) |  |
| NA | 1,868 (0.5%) | 909 (0.9%) |  |
| Diabetes mellitus, N (%) |  |  | <0.001 |
| No | 381,202 (93.9%) | 88,962 (92.2%) |  |
| Yes | 24,705 (6.1%) | 5,974 (6.2%) |  |
| NA | 0 (0.0%) | 1,512 (1.6%) |  |
| Hypertension, N (%) |  |  | <0.001 |
| No | 186531 (46.0%) | 44,279 (45.9%) |  |
| Yes | 219,376 (54.0%) | 51,210 (53.1%) |  |
| NA | 0 (0.0%) | 959 (1.0%) |  |
| Cardiovascular diseases, N (%) |  |  | 0.081 |
| No | 374,152 (92.18%) | 88,740 (92.01%) |  |
| Yes | 31,755 (7.82%) | 7,708 (7.99%) |  |
| Lipid lowering drug, N (%) |  |  | <0.001 |
| No | 334,614 (82.4%) | 76,153 (79.0%) |  |
| Yes | 70,879 (17.5%) | 15,993 (16.6%) |  |
| NA | 414 (0.1%) | 4,302 (4.5%) |  |
| Antihypertensives, N (%) |  |  | <0.001 |
| No | 320,854 (79.0%) | 72,805 (75.5%) |  |
| Yes | 84,639 (20.9%) | 19,341 (20.1%) |  |
| NA | 414 (0.1%) | 4,302 (4.5%) |  |
| Antidiabetic drugs, N (%) |  |  | <0.001 |
| No | 390,837 (96.3%) | 90,524 (93.9%) |  |
| Yes | 14,912 (3.7%) | 3,831 (4.0%) |  |
| NA | 158 (0.0%) | 2,093 (2.2%) |  |
| Family history of cancer, N (%) |  |  | 0.002 |
| No | 282,863 (69.69%) | 67,705 (70.20%) |  |
| Yes | 123,044 (30.31%) | 28,743 (29.80%) |  |
| Smoking status, N (%) |  |  | <0.001 |
| Never | 161,714 (39.8%) | 39,120 (40.6%) |  |
| Previous | 199,643 (49.2%) | 46,366 (48.1%) |  |
| Current | 42,525 (10.5%) | 10,038 (10.4%) |  |
| NA | 2,025 (0.5%) | 924 (1.0%) |  |
| Alcohol intake frequency, N (%) |  |  | <0.001 |
| Never | 32,283 (8.0%) | 8,338 (8.6%) |  |
| Special occasions only | 46,253 (11.4%) | 11,733 (12.2%) |  |
| One to three times a month | 45,112 (11.1%) | 10,723 (11.1%) |  |
| Once or twice a week | 104,826 (25.8%) | 24,429 (25.3%) |  |
| Three or four times a week | 93,835 (23.1%) | 21,577 (22.4%) |  |
| Daily or almost daily | 82,748 (20.4%) | 18,997 (19.7%) |  |
| NA | 850 (0.2%) | 651 (0.7%) |  |

| **Table S3. Information on missing covariates among 369,447 UK Biobank participants.** | | | |
| --- | --- | --- | --- |
| **Variable** | **Missing, n (%)** | **Global LR χ² (df)** | **P value** |
| **Any covariate** | **89,839 (24.32%)** | **5235.7 (6)** | **<0.001** |
| Race and ethnicity | 1,738 (0.47%) | 180.1 (6) | <0.001 |
| Townsend Deprivation Index | 457 (0.12%) | 69.2 (6) | <0.001 |
| Body mass index | 747 (0.20%) | 237.6 (6) | <0.001 |
| Smoking status | 1,828 (0.49%) | 245.2 (6) | <0.001 |
| Antihypertensive medication use | 396 (0.11%) | 292.8 (6) | <0.001 |
| Lipid-lowering medication use | 396 (0.11%) | 292.8 (6) | <0.001 |
| Antidiabetic medication use | 155 (0.04%) | 124.4 (6) | <0.001 |
| Alcohol intake frequency | 805 (0.22%) | 303.6 (6) | <0.001 |
| Diet score | 13,734 (3.72%) | 1057.5 (6) | <0.001 |
| Total MET-min/week | 82,987 (22.46%) | 5928.9 (6) | <0.001 |

LR: likelihood ratio; For each incomplete covariate, a binary missingness indicator (1 = missing; 0 = observed) was modeled using age, sex, T2DM, hypertension, CVD, and family history of cancer, all of which were fully observed. The global LR test compared the full model with an intercept-only model. Little’s test based on age, Townsend Deprivation Index, body mass index, diet score, and total MET-min/week identified 14 missing-data patterns and rejected MCAR (χ²=9,304, df=40, P<0.001).

| Table S4. Variables included in the multiple imputation model. | |
| --- | --- |
| Imputed Variable | Predictors Used |
| Race and ethnicity | Age, Sex, TDI, BMI, Smoking status, DM, Hypertension, CVD, Antihypertensive, Lipid lowering drug, Antidiabetic drugs, Family history of cancer, Alcohol intake frequency, Diet score, MET |
| TDI | Age, Sex, Race and ethnicity, BMI, Smoking status, DM, Hypertension, CVD, Antihypertensive, Lipid lowering drug, Antidiabetic drugs, Family history of cancer, Alcohol intake frequency, Diet score, MET |
| BMI | Age, Sex, Race and ethnicity, TDI, Smoking status, DM, Hypertension, CVD, Antihypertensive, Lipid lowering drug, Antidiabetic drugs, Family history of cancer, Alcohol intake frequency, Diet score, MET |
| Smoking status | Age, Sex, Race and ethnicity, TDI, BMI, DM, Hypertension, CVD, Antihypertensive, Lipid lowering drug, Antidiabetic drugs, Family history of cancer, Alcohol intake frequency, Diet score, MET |
| Alcohol intake frequency | Age, Sex, Race and ethnicity, TDI, BMI, Smoking status, DM, Hypertension, CVD, Antihypertensive, Lipid lowering drug, Antidiabetic drugs, Family history of cancer, Diet score, MET |
| Antihypertensive | Age, Sex, Race and ethnicity, TDI, BMI, Smoking status, DM, Hypertension, CVD, Lipid lowering drug, Antidiabetic drugs, Family history of cancer, Alcohol intake frequency, Diet score, MET |
| Lipid lowering drug | Age, Sex, Race and ethnicity, TDI, BMI, Smoking status, DM, Hypertension, CVD, Antihypertensive, Antidiabetic drugs, Family history of cancer, Alcohol intake frequency, Diet score, MET |
| Antidiabetic drugs | Age, Sex, Race and ethnicity, TDI, BMI, Smoking status, DM, Hypertension, CVD, Antihypertensive, Lipid lowering drug, Family history of cancer, Alcohol intake frequency, Diet score, MET |
| Diet score | Age, Sex, Race and ethnicity, TDI, BMI, Smoking status, DM, Hypertension, CVD, Antihypertensive, Lipid lowering drug, Antidiabetic drugs, Family history of cancer, Alcohol intake frequency, MET |
| MET | Age, Sex, Race and ethnicity, TDI, BMI, Smoking status, DM, Hypertension, CVD, Antihypertensive, Lipid lowering drug, Antidiabetic drugs, Family history of cancer, Alcohol intake frequency, Diet score |

TDI: Townsend Deprivation Index; BMI: Body mass index; MET: Metabolic equivalent of task

| Table S5. Sensitivity analysis of associations of per-SD increases in eGDR, TG/HDL-C, and TyG with GI cancer mortality after excluding participants who died from GI cancer within the first 2 years. | | | | | | |
| --- | --- | --- | --- | --- | --- | --- |
| **Types** | **eGDR** | | **TG/HDL-C** | | **TyG** | |
|  | **sHR (95% CI)** | ***P*** | **sHR (95% CI)** | ***P*** | **sHR (95% CI)** | ***P*** |
| EC | 0.65 (0.54-0.78) | <0.001 | 0.98 (0.92-1.06) | 0.64 | 1.04 (0.96-1.12) | 0.37 |
| EAC | 0.74 (0.49-1.10) | 0.13 | 1.00 (0.92-1.10) | 0.93 | 1.10 (0.98-1.23) | 0.11 |
| ESCC | 0.63 (0.47-0.85) | 0.002 | 0.97 (0.74-1.27) | 0.81 | 0.92 (0.73-1.17) | 0.49 |
| GC | 0.71 (0.51-0.99) | 0.041 | 1.04 (0.94-1.15) | 0.42 | 0.99 (0.88-1.12) | 0.9 |
| CRC | 0.68 (0.57-0.80) | <0.001 | 1.04 (0.99-1.10) | 0.14 | 1.03 (0.97-1.10) | 0.35 |
| LC | 0.67 (0.54-0.82) | <0.001 | 1.12 (1.04-1.20) | 0.003 | 1.13 (1.03-1.25) | 0.01 |
| PC | 0.69 (0.59-0.81) | <0.001 | 1.02 (0.96-1.08) | 0.53 | 1.05 (0.98-1.12) | 0.14 |
| GI | 0.68 (0.61-0.75) | <0.001 | 1.04 (1.01-1.07) | 0.012 | 1.05 (1.01-1.09) | 0.006 |

GI cancer: gastrointestinal cancer; EC: esophageal cancer; EAC: esophageal adenocarcinoma; ESCC: esophageal squamous cell carcinoma; GC: gastric cancer; CRC: colorectal cancer; LC: liver cancer; PC: pancreatic cancer; SD: standard deviation; sHR: subdistribution hazard ratio; Models were adjusted for age, sex, race and ethnicity, BMI, TDI, total MET-min/week, smoking status, alcohol consumption frequency, antihypertensive, lipid-lowering, and glucose-lowering medication use, T2DM, hypertension, CVD, family history of cancer, and diet score.

| Table S6. Sensitivity analysis of associations of per-SD increases in eGDR, TG/HDL-C, and TyG with GI cancer mortality in the complete-case analysis. | | | | | | |
| --- | --- | --- | --- | --- | --- | --- |
| **Types** | **eGDR** | | **TG/HDL-C** | | **TyG** | |
|  | **sHR (95% CI)** | ***P*** | **sHR (95% CI)** | ***P*** | **sHR (95% CI)** | ***P*** |
| EC | 0.64 (0.53-0.78) | <0.001 | 0.94 (0.87-1.03) | 0.18 | 0.98 (0.90-1.08) | 0.69 |
| EAC | 0.66 (0.47-0.94) | 0.021 | 1.00 (0.91-1.10) | 0.98 | 1.10 (0.96-1.24) | 0.16 |
| ESCC | 0.79 (0.46-1.35) | 0.38 | 0.69 (0.45-1.06) | 0.091 | 0.68 (0.50-0.92) | 0.012 |
| GC | 0.65 (0.48-0.88) | 0.006 | 1.05 (0.95-1.16) | 0.36 | 1.04 (0.91-1.19) | 0.53 |
| CRC | 0.63 (0.54-0.74) | <0.001 | 1.02 (0.95-1.09) | 0.59 | 1.02 (0.95-1.09) | 0.64 |
| LC | 0.62 (0.53-0.79) | <0.001 | 1.10 (1.01-1.20) | 0.031 | 1.14 (1.02-1.27) | 0.018 |
| PC | 0.71 (0.60-0.84) | <0.001 | 1.02 (0.95-1.09) | 0.65 | 1.06 (0.99-1.14) | 0.085 |
| GI | 0.66 (0.59-0.74) | <0.001 | 1.03 (1.01-1.06) | 0.02 | 1.04 (1.01-1.08) | 0.026 |

GI cancer: gastrointestinal cancer; EC: esophageal cancer; EAC: esophageal adenocarcinoma; ESCC: esophageal squamous cell carcinoma; GC: gastric cancer; CRC: colorectal cancer; LC: liver cancer; PC: pancreatic cancer; SD: standard deviation; sHR: subdistribution hazard ratio; Models were adjusted for age, sex, race and ethnicity, BMI, TDI, total MET-min/week, smoking status, alcohol consumption frequency, antihypertensive, lipid-lowering, and glucose-lowering medication use, T2DM, hypertension, CVD, family history of cancer, and diet score.

| Table S7. Sensitivity analysis of associations of per-SD increases in eGDR, TG/HDL-C, and TyG with GI cancer mortality using multiple imputation by chained equations with predictive mean matching (MICE-PMM). | | | | | | |
| --- | --- | --- | --- | --- | --- | --- |
| **Types** | **eGDR** | | **TG/HDL-C** | | **TyG** | |
|  | **sHR (95% CI)** | ***P*** | **sHR (95% CI)** | ***P*** | **sHR (95% CI)** | ***P*** |
| EC | 0.65 (0.54-0.79) | <0.001 | 0.97 (0.91-1.04) | 0.435 | 1.02 (0.95-1.10) | 0.59 |
| EAC | 0.75 (0.51-1.10) | 0.143 | 1.00 (0.92-1.09) | 0.989 | 1.09 (0.98-1.22) | 0.116 |
| ESCC | 0.63 (0.46-0.86) | 0.004 | 0.90 (0.67-1.21) | 0.485 | 0.85 (0.67-1.07) | 0.17 |
| GC | 0.71 (0.51-0.99) | 0.045 | 1.03 (0.93-1.13) | 0.567 | 1.00 (0.89-1.12) | 0.965 |
| CRC | 0.66 (0.57-0.78) | <0.001 | 1.04 (0.98-1.10) | 0.16 | 1.03 (0.97-1.10) | 0.304 |
| LC | 0.65 (0.53-0.78) | <0.001 | 1.12 (1.04-1.20) | 0.002 | 1.14 (1.04-1.25) | 0.005 |
| PC | 0.68 (0.58-0.80) | <0.001 | 1.01 (0.96-1.07) | 0.646 | 1.05 (0.98-1.12) | 0.141 |
| GI | 0.67 (0.60-0.74) | <0.001 | 1.03 (1.00-1.06) | 0.027 | 1.05 (1.01-1.08) | 0.006 |

GI cancer: gastrointestinal cancer; EC: esophageal cancer; EAC: esophageal adenocarcinoma; ESCC: esophageal squamous cell carcinoma; GC: gastric cancer; CRC: colorectal cancer; LC: liver cancer; PC: pancreatic cancer; SD: standard deviation; sHR: subdistribution hazard ratio; MICE-PMM: multiple imputation by chained equations with predictive mean matching; Models were adjusted for age, sex, race and ethnicity, BMI, TDI, total MET-min/week, smoking status, alcohol consumption frequency, antihypertensive, lipid-lowering, and glucose-lowering medication use, T2DM, hypertension, CVD, family history of cancer, and diet score.

| Table S8. Sensitivity analysis of associations of per-SD increases in eGDR, TG/HDL-C, and TyG with GI cancer mortality with additional adjustment for baseline proton pump inhibitor use. | | | | | | |
| --- | --- | --- | --- | --- | --- | --- |
| **Types** | **eGDR** | | **TG/HDL-C** | | **TyG** | |
|  | **sHR (95% CI)** | ***P*** | **sHR (95% CI)** | ***P*** | **sHR (95% CI)** | ***P*** |
| EC | 0.68 (0.56-0.82) | <0.001 | 0.97 (0.91-1.04) | 0.45 | 1.02 (0.95-1.10) | 0.61 |
| EAC | 0.79 (0.53-1.16) | 0.22 | 1.00 (0.92-1.09) | 0.95 | 1.09 (0.98-1.22) | 0.11 |
| ESCC | 0.64 (0.48-0.86) | 0.003 | 0.89 (0.66-1.20) | 0.46 | 0.84 (0.67-1.07) | 0.16 |
| GC | 0.70 (0.51-0.97) | 0.031 | 1.04 (0.94-1.14) | 0.49 | 1.00 (0.89-1.13) | 0.96 |
| CRC | 0.66 (0.56-0.77) | <0.001 | 1.04 (0.98-1.10) | 0.17 | 1.03 (0.97-1.10) | 0.30 |
| LC | 0.66 (0.55-0.81) | <0.001 | 1.12 (1.05-1.21) | 0.002 | 1.14 (1.04-1.25) | 0.007 |
| PC | 0.68 (0.58-0.79) | <0.001 | 1.02 (0.96-1.08) | 0.54 | 1.05 (0.99-1.12) | 0.11 |
| GI | 0.67 (0.61-0.75) | <0.001 | 1.04 (1.01-1.07) | 0.019 | 1.05 (1.02-1.08) | 0.005 |

GI cancer: gastrointestinal cancer; EC: esophageal cancer; EAC: esophageal adenocarcinoma; ESCC: esophageal squamous cell carcinoma; GC: gastric cancer; CRC: colorectal cancer; LC: liver cancer; PC: pancreatic cancer; SD: standard deviation; sHR: subdistribution hazard ratio; Models were adjusted for age, sex, race and ethnicity, BMI, TDI, total MET-min/week, smoking status, alcohol consumption frequency, antihypertensive, lipid-lowering, and glucose-lowering medication use, T2DM, hypertension, CVD, family history of cancer, and diet score. Models were additionally adjusted for baseline proton pump inhibitor use.

| Table S9. Sensitivity analysis of associations of per-SD increases in eGDR, TG/HDL-C, and TyG with GI cancer mortality using cause-specific Cox proportional hazards models. | | | | | | |
| --- | --- | --- | --- | --- | --- | --- |
| **Types** | **eGDR** | | **TG/HDL-C** | | **TyG** | |
|  | **HR (95% CI)** | ***P*** | **HR (95% CI)** | ***P*** | **HR (95% CI)** | ***P*** |
| EC | 0.64 (0.51-0.79) | <0.001 | 0.97 (0.90-1.04) | 0.372 | 1.02 (0.94-1.10) | 0.692 |
| EAC | 0.73 (0.48-1.09) | 0.126 | 1.00 (0.90-1.10) | 0.977 | 1.09 (0.97-1.23) | 0.159 |
| ESCC | 0.59 (0.40-0.88) | 0.009 | 0.89 (0.71-1.11) | 0.308 | 0.85 (0.69-1.03) | 0.096 |
| GC | 0.69 (0.49-0.98) | 0.04 | 1.03 (0.93-1.13) | 0.596 | 0.99 (0.89-1.11) | 0.918 |
| CRC | 0.65 (0.54-0.77) | <0.001 | 1.04 (0.98-1.09) | 0.184 | 1.03 (0.97-1.09) | 0.335 |
| LC | 0.63 (0.51-0.78) | <0.001 | 1.11 (1.04-1.19) | 0.001 | 1.13 (1.04-1.24) | 0.005 |
| PC | 0.65 (0.55-0.77) | <0.001 | 1.02 (0.96-1.08) | 0.56 | 1.05 (0.99-1.12) | 0.131 |
| GI | 0.65 (0.59-0.71) | <0.001 | 1.03 (1.00-1.06) | 0.033 | 1.05 (1.01-1.08) | 0.008 |

GI cancer: gastrointestinal cancer; EC: esophageal cancer; EAC: esophageal adenocarcinoma; ESCC: esophageal squamous cell carcinoma; GC: gastric cancer; CRC: colorectal cancer; LC: liver cancer; PC: pancreatic cancer; SD: standard deviation; HR: cause-specific hazard ratio; Models were adjusted for age, sex, race and ethnicity, BMI, TDI, total MET-min/week, smoking status, alcohol consumption frequency, antihypertensive, lipid-lowering, and glucose-lowering medication use, T2DM, hypertension, CVD, family history of cancer, and diet score.

| Table S10. Sensitivity analysis of the association of a per-SD increase in eGDR with GI cancer mortality after excluding participants with T2DM. | | |
| --- | --- | --- |
| **Type** | **sHR (95% CI)** | ***P*** |
| EC | 0.58 (0.42-0.81) | 0.001 |
| EAC | 0.70 (0.43-1.16) | 0.171 |
| ESCC | 0.64 (0.30-1.40) | 0.269 |
| GC | 0.52 (0.34-0.81) | 0.004 |
| CRC | 0.61 (0.48-0.78) | <0.001 |
| LC | 0.43 (0.28-0.65) | <0.001 |
| PC | 0.71 (0.55-0.92) | 0.01 |
| GI | 0.60 (0.52-0.68) | <0.001 |

GI cancer: gastrointestinal cancer; EC: esophageal cancer; EAC: esophageal adenocarcinoma; ESCC: esophageal squamous cell carcinoma; GC: gastric cancer; CRC: colorectal cancer; LC: liver cancer; PC: pancreatic cancer; SD: standard deviation; sHR: subdistribution hazard ratio; Models were adjusted for age, sex, race and ethnicity, BMI, TDI, total MET-min/week, smoking status, alcohol consumption frequency, antihypertensive and lipid-lowering medication use, hypertension, CVD, family history of cancer, and diet score.

Table S11. Sensitivity analysis of the associations of per-SD increases in eGDR, TG/HDL-C, and TyG with gastrointestinal cancer mortality after excluding potentially overlapping metabolic covariates.

| **Types** | **eGDR** | | **TG/HDL-C** | | **TyG** | |
| --- | --- | --- | --- | --- | --- | --- |
|  | **sHR (95% CI)** | ***P*** | **sHR (95% CI)** | ***P*** | **sHR (95% CI)** | ***P*** |
| EC | 0.75 (0.69-0.82) | <0.001 | 1.02 (0.96-1.09) | 0.494 | 1.09 (1.02-1.18) | 0.016 |
| EAC | 0.66 (0.58-0.76) | <0.001 | 1.07 (1.00-1.16) | 0.06 | 1.21 (1.09-1.35) | <0.001 |
| ESCC | 0.97 (0.78-1.20) | 0.764 | 0.84 (0.63-1.12) | 0.235 | 0.81 (0.66-0.99) | 0.04 |
| GC | 0.87 (0.77-0.99) | 0.035 | 1.07 (0.98-1.17) | 0.11 | 1.06 (0.95-1.19) | 0.275 |
| CRC | 0.88 (0.82-0.93) | <0.001 | 1.07 (1.01-1.12) | 0.013 | 1.07 (1.01-1.14) | 0.016 |
| LC | 0.68 (0.61-0.76) | <0.001 | 1.20 (1.13-1.28) | <0.001 | 1.34 (1.23-1.47) | <0.001 |
| PC | 0.80 (0.75-0.86) | <0.001 | 1.07 (1.02-1.13) | 0.008 | 1.14 (1.07-1.21) | <0.001 |
| GI | 0.80 (0.77-0.83) | <0.001 | 1.08 (1.06-1.11) | <0.001 | 1.13 (1.09-1.17) | <0.001 |

eGDR, estimated glucose disposal rate; TG/HDL-C, triglyceride-to-high-density lipoprotein cholesterol ratio; TyG, triglyceride-glucose index; sHR, subdistribution hazard ratio; CI, confidence interval; SD, standard deviation. Models were adjusted for age, sex, ethnicity, Townsend Deprivation Index, smoking status, alcohol consumption, physical activity, dietary score, and family history of cancer.

Table S12. Sensitivity analysis of the associations of per-SD increases in eGDR, TG/HDL-C, and TyG with gastrointestinal cancer mortality after excluding lifestyle covariates.

| **Types** | **eGDR** | | **TG/HDL-C** | | **TyG** | |
| --- | --- | --- | --- | --- | --- | --- |
|  | **sHR (95% CI)** | ***P*** | **sHR (95% CI)** | ***P*** | **sHR (95% CI)** | ***P*** |
| EC | 0.59 (0.49-0.70) | <0.001 | 1.00 (0.93-1.07) | 0.978 | 1.05 (0.97-1.13) | 0.252 |
| EAC | 0.66 (0.47-0.92) | 0.016 | 1.03 (0.95-1.12) | 0.483 | 1.12 (1.00-1.25) | 0.045 |
| ESCC | 0.55 (0.42-0.72) | <0.001 | 0.92 (0.68-1.24) | 0.576 | 0.87 (0.69-1.11) | 0.264 |
| GC | 0.69 (0.50-0.93) | 0.016 | 1.05 (0.95-1.15) | 0.332 | 1.01 (0.90-1.14) | 0.867 |
| CRC | 0.64 (0.55-0.74) | <0.001 | 1.04 (0.99-1.10) | 0.142 | 1.04 (0.98-1.10) | 0.219 |
| LC | 0.59 (0.50-0.71) | <0.001 | 1.14 (1.07-1.22) | <0.001 | 1.17 (1.07-1.28) | <0.001 |
| PC | 0.64 (0.55-0.75) | <0.001 | 1.03 (0.97-1.09) | 0.401 | 1.06 (1.00-1.13) | 0.065 |
| GI | 0.63 (0.57-0.70) | <0.001 | 1.05 (1.02-1.08) | 0.002 | 1.06 (1.03-1.10) | <0.001 |

eGDR, estimated glucose disposal rate; TG/HDL-C, triglyceride-to-high-density lipoprotein cholesterol ratio; TyG, triglyceride-glucose index; sHR, subdistribution hazard ratio; CI, confidence interval; SD, standard deviation. Models were adjusted for age, sex, ethnicity, BMI, Townsend Deprivation Index, type 2 diabetes, hypertension, cardiovascular disease, family history of cancer, and related medication use.

| Table S13. Associations of per-SD increases in eGDR, TG/HDL-C, and TyG with GI cancer mortality stratified by sex. | | | | | | | | | |
| --- | --- | --- | --- | --- | --- | --- | --- | --- | --- |
| **Types** | **eGDR** | | | **TG/HDL-C** | | | **TyG** | | |
|  | **Women** | **Men** | ***P* for interaction** | **Women** | **Men** | ***P* for interaction** | **Women** | **Men** | ***P* for interaction** |
|  | **sHR (95% CI)** | **sHR (95% CI)** |  | **sHR (95% CI)** | **sHR (95% CI)** |  | **sHR (95% CI)** | **sHR (95% CI)** |  |
| EC | **0.42 (0.28-0.63)** | **0.73 (0.55-0.97)** | **0.031** | 1.00 (0.82-1.21) | 0.97 (0.90-1.05) | 0.812 | 1.06 (0.90-1.25) | 1.01 (0.93-1.11) | 0.624 |
| EAC | **0.37 (0.17-0.82)** | 0.84 (0.54-1.32) | 0.079 | 1.12 (0.86-1.44) | 1.01 (0.92-1.10) | 0.459 | 1.27 (0.94-1.72) | 1.07 (0.96-1.21) | 0.301 |
| ESCC | **0.32 (0.17-0.58)** | 0.99 (0.33-2.91) | 0.072 | 0.96 (0.63-1.49) | 0.83 (0.55-1.26) | 0.633 | 0.96 (0.71-1.31) | 0.74 (0.52-1.06) | 0.276 |
| GC | 1.05 (0.51-2.14) | **0.62 (0.48-0.8)** | 0.176 | 1.05 (0.86-1.29) | 1.03 (0.93-1.15) | 0.868 | 1.10 (0.90-1.34) | 0.97 (0.84-1.12) | 0.321 |
| CRC | **0.67 (0.49-0.92)** | **0.65 (0.54-0.78)** | 0.887 | 1.00 (0.90-1.12) | 1.05 (0.98-1.11) | 0.499 | 1.03 (0.94-1.13) | 1.03 (0.95-1.12) | 0.951 |
| LC | **0.56 (0.35-0.9)** | **0.66 (0.53-0.83)** | 0.521 | 1.18 (0.98-1.42) | **1.11 (1.03-1.19)** | 0.552 | 1.13 (0.96-1.33) | **1.15 (1.02-1.28)** | 0.87 |
| PC | **0.73 (0.53-0.99)** | **0.62 (0.52-0.74)** | 0.386 | 1.10 (0.98-1.23) | 1.00 (0.93-1.07) | 0.141 | 1.08 (0.98-1.20) | 1.04 (0.97-1.13) | 0.594 |
| GI | **0.66 (0.55-0.79)** | **0.65 (0.58-0.74)** | 0.927 | 1.06 (0.99-1.13) | 1.03 (1.00-1.07) | 0.507 | **1.07 (1.01-1.13)** | **1.04 (1.00-1.09)** | 0.55 |

GI: gastrointestinal cancer; EC: esophageal cancer; EAC: esophageal adenocarcinoma; ESCC: esophageal squamous cell carcinoma; GC: gastric cancer; CRC: colorectal cancer; LC: liver cancer; PC: pancreatic cancer; CI: confidence interval; SD: standard deviation; sHR: subdistribution hazard ratio. Fully adjusted Fine–Gray models included the Model 2 covariates specified in the main manuscript, excluding the stratification variable.

| Table S14. Associations of per-SD increases in eGDR, TG/HDL-C, and TyG with GI cancer mortality stratified by age. | | | | | | | | | |
| --- | --- | --- | --- | --- | --- | --- | --- | --- | --- |
| **Types** | **eGDR** | | | **TG/HDL-C** | | | **TyG** | | |
|  | **<60 years** | **≥60 years** | ***P* for interaction** | **<60 years** | **≥60 years** | ***P* for interaction** | **<60 years** | **≥60 years** | ***P* for interaction** |
|  | **sHR (95% CI)** | **sHR (95% CI)** |  | **sHR (95% CI)** | **sHR (95% CI)** |  | **sHR (95% CI)** | **sHR (95% CI)** |  |
| EC | **0.36 (0.24-0.55)** | 0.78 (0.57-1.06) | **0.004** | 1.04 (0.93-1.15) | 0.94 (0.86-1.03) | 0.185 | 1.04 (0.92-1.18) | 1.01 (0.92-1.12) | 0.759 |
| EAC | 0.59 (0.29-1.20) | 0.82 (0.50-1.35) | 0.445 | 0.99 (0.87-1.14) | 1.03 (0.92-1.15) | 0.687 | 1.07 (0.91-1.26) | 1.11 (0.96-1.29) | 0.71 |
| ESCC | **0.17 (0.09-0.33)** | 0.96 (0.41-2.28) | **0.002** | 1.05 (0.69-1.60) | 0.78 (0.54-1.12) | 0.29 | 0.79 (0.52-1.19) | 0.88 (0.65-1.17) | 0.682 |
| GC | **0.34 (0.19-0.62)** | 0.94 (0.59-1.49) | **0.008** | 1.05 (0.92-1.21) | 1.02 (0.90-1.16) | 0.769 | 1.10 (0.89-1.36) | 0.96 (0.83-1.10) | 0.283 |
| CRC | **0.52 (0.38-0.71)** | **0.72 (0.58-0.90)** | 0.085 | 1.06 (0.97-1.16) | 1.03 (0.96-1.10) | 0.586 | 1.04 (0.94-1.15) | 1.03 (0.96-1.11) | 0.911 |
| LC | 0.57 (0.32-1.02) | **0.67 (0.54-0.82)** | 0.609 | 1.11 (0.96-1.27) | **1.13 (1.04-1.22)** | 0.816 | 1.07 (0.92-1.24) | **1.18 (1.05-1.32)** | 0.32 |
| PC | **0.48 (0.32-0.70)** | **0.71 (0.59-0.86)** | 0.069 | 1.06 (0.97-1.16) | 1.01 (0.93-1.08) | 0.392 | 1.11 (0.99-1.24) | 1.03 (0.96-1.11) | 0.284 |
| GI | **0.47 (0.39-0.57)** | **0.72 (0.65-0.81)** | **<0.001** | **1.06 (1.01-1.11)** | 1.03 (0.99-1.07) | 0.35 | **1.06 (1.00-1.12)** | **1.05 (1.01-1.09)** | 0.735 |

GI: gastrointestinal cancer; EC: esophageal cancer; EAC: esophageal adenocarcinoma; ESCC: esophageal squamous cell carcinoma; GC: gastric cancer; CRC: colorectal cancer; LC: liver cancer; PC: pancreatic cancer; CI: confidence interval; SD: standard deviation; sHR: subdistribution hazard ratio. Fully adjusted Fine–Gray models included the Model 2 covariates specified in the main manuscript, excluding the stratification variable.

| Table S15. Associations of per-SD increases in eGDR, TG/HDL-C, and TyG with GI cancer mortality stratified by BMI. | | | | | | | | | |
| --- | --- | --- | --- | --- | --- | --- | --- | --- | --- |
| **Types** | **eGDR** | | | **TG/HDL-C** | | | **TyG** | | |
|  | **<30 kg/m²** | **≥30 kg/m²** | ***P* for interaction** | **<30 kg/m²** | **≥30 kg/m²** | ***P* for interaction** | **<30 kg/m²** | **≥30 kg/m²** | ***P* for interaction** |
|  | **sHR (95% CI)** | **sHR (95% CI)** |  | **sHR (95% CI)** | **sHR (95% CI)** |  | **sHR (95% CI)** | **sHR (95% CI)** |  |
| EC | **0.62 (0.51-0.75)** | 0.71 (0.47-1.08) | 0.539 | 1.02 (0.93-1.12) | 0.95 (0.85-1.06) | 0.354 | 1.05 (0.96-1.16) | 1.01 (0.89-1.15) | 0.613 |
| EAC | 0.72 (0.47-1.11) | 0.79 (0.43-1.47) | 0.809 | 1.07 (0.96-1.19) | 0.94 (0.82-1.08) | 0.15 | 1.10 (0.95-1.27) | 1.06 (0.90-1.26) | 0.781 |
| ESCC | **0.55 (0.41-0.74)** | 0.51 (0.14-1.84) | 0.91 | 1.10 (0.85-1.42) | 0.45 (0.20-1.04) | 0.045 | 1.06 (0.83-1.35) | **0.42 (0.24-0.72)** | **0.002** |
| GC | 0.76 (0.52-1.11) | 0.64 (0.39-1.05) | 0.559 | 1.08 (0.95-1.23) | 1.02 (0.89-1.16) | 0.491 | 1.03 (0.88-1.20) | 1.02 (0.85-1.23) | 0.953 |
| CRC | **0.65 (0.54-0.78)** | **0.66 (0.47-0.93)** | 0.892 | 1.03 (0.95-1.10) | 1.05 (0.96-1.13) | 0.735 | 1.01 (0.94-1.09) | 1.07 (0.97-1.2) | 0.381 |
| LC | **0.69 (0.53-0.90)** | **0.56 (0.38-0.84)** | 0.415 | **1.15 (1.03-1.28)** | **1.11 (1.01-1.21)** | 0.638 | **1.16 (1.02-1.31)** | 1.15 (1.00-1.32) | 0.902 |
| PC | **0.73 (0.59-0.91)** | **0.51 (0.37-0.71)** | 0.066 | 1.05 (0.97-1.13) | 0.98 (0.90-1.08) | 0.315 | 1.07 (0.99-1.15) | 1.03 (0.92-1.15) | 0.597 |
| GI | **0.67 (0.59-0.76)** | **0.61 (0.51-0.72)** | 0.362 | **1.05 (1.01-1.10)** | 1.02 (0.98-1.07) | 0.286 | **1.06 (1.01-1.10)** | **1.06 (1.00-1.12)** | 0.955 |

GI: gastrointestinal cancer; EC: esophageal cancer; EAC: esophageal adenocarcinoma; ESCC: esophageal squamous cell carcinoma; GC: gastric cancer; CRC: colorectal cancer; LC: liver cancer; PC: pancreatic cancer; CI: confidence interval; SD: standard deviation; sHR: subdistribution hazard ratio. Fully adjusted Fine–Gray models included the Model 2 covariates specified in the main manuscript, excluding the stratification variable. BMI: body mass index.

| Table S16. Associations of per-SD increases in eGDR, TG/HDL-C, and TyG with GI cancer mortality stratified by TDI. | | | | | | | | | |
| --- | --- | --- | --- | --- | --- | --- | --- | --- | --- |
| **Types** | **eGDR** | | | **TG/HDL-C** | | | **TyG** | | |
|  | **<−1.31** | **≥−1.31** | ***P* for interaction** | **<−1.31** | **≥−1.31** | ***P* for interaction** | **<−1.31** | **≥−1.31** | ***P* for interaction** |
|  | **sHR (95% CI)** | **sHR (95% CI)** |  | **sHR (95% CI)** | **sHR (95% CI)** |  | **sHR (95% CI)** | **sHR (95% CI)** |  |
| EC | **0.57 (0.47-0.70)** | 0.72 (0.49-1.07) | 0.292 | 1.06 (0.97-1.16) | 0.90 (0.81-1.01) | 0.025 | **1.12 (1.01-1.25)** | 0.94 (0.84-1.05) | **0.021** |
| EAC | 0.70 (0.46-1.05) | 0.77 (0.42-1.42) | 0.788 | 1.07 (0.97-1.19) | 0.95 (0.82-1.09) | 0.168 | **1.17 (1.01-1.36)** | 1.02 (0.87-1.20) | 0.225 |
| ESCC | **0.47 (0.32-0.69)** | 0.78 (0.32-1.90) | 0.3 | 1.12 (0.83-1.51) | 0.73 (0.42-1.27) | 0.178 | 1.07 (0.75-1.52) | **0.72 (0.52-0.98)** | 0.1 |
| GC | **0.63 (0.41-0.97)** | 0.77 (0.46-1.30) | 0.561 | **1.15 (1.03-1.28)** | 0.93 (0.79-1.08) | **0.026** | 1.15 (0.98-1.35) | 0.88 (0.74-1.05) | 0.028 |
| CRC | **0.69 (0.57-0.85)** | **0.57 (0.42-0.78)** | 0.3 | 1.04 (0.97-1.12) | 1.04 (0.96-1.13) | 0.99 | 1.01 (0.94-1.09) | 1.06 (0.97-1.17) | 0.429 |
| LC | **0.62 (0.51-0.76)** | 0.71 (0.46-1.08) | 0.59 | **1.13 (1.03-1.24)** | 1.10 (0.99-1.23) | 0.709 | **1.16 (1.03-1.31)** | 1.11 (0.96-1.28) | 0.626 |
| PC | **0.65 (0.55-0.77)** | **0.68 (0.49-0.94)** | 0.803 | 1.02 (0.94-1.11) | 1.02 (0.94-1.11) | 0.994 | 1.04 (0.96-1.13) | 1.08 (0.98-1.18) | 0.612 |
| GI | **0.64 (0.56-0.72)** | **0.68 (0.58-0.8)** | 0.509 | **1.06 (1.02-1.10)** | 1.01 (0.97-1.06) | 0.108 | **1.07 (1.02-1.12)** | 1.03 (0.98-1.08) | 0.275 |

GI: gastrointestinal cancer; EC: esophageal cancer; EAC: esophageal adenocarcinoma; ESCC: esophageal squamous cell carcinoma; GC: gastric cancer; CRC: colorectal cancer; LC: liver cancer; PC: pancreatic cancer; CI: confidence interval; SD: standard deviation; sHR: subdistribution hazard ratio. Fully adjusted Fine–Gray models included the Model 2 covariates specified in the main manuscript, excluding the stratification variable. TDI: Townsend Deprivation Index.

| Table S17. Associations of per-SD increases in eGDR, TG/HDL-C, and TyG with GI cancer mortality stratified by CRP. | | | | | | | | | |
| --- | --- | --- | --- | --- | --- | --- | --- | --- | --- |
| **Types** | **eGDR** | | | **TG/HDL-C** | | | **TyG** | | |
|  | **<2.57 mg/L** | **≥2.57 mg/L** | ***P* for interaction** | **<2.57 mg/L** | **≥2.57 mg/L** | ***P* for interaction** | **<2.57 mg/L** | **≥2.57 mg/L** | ***P* for interaction** |
|  | **sHR (95% CI)** | **sHR (95% CI)** |  | **sHR (95% CI)** | **sHR (95% CI)** |  | **sHR (95% CI)** | **sHR (95% CI)** |  |
| EC | **0.58 (0.4-0.84)** | **0.71 (0.54-0.92)** | 0.382 | 0.98 (0.89-1.07) | 0.98 (0.88-1.09) | 0.954 | 1.02 (0.92-1.12) | 1.03 (0.9-1.17) | 0.881 |
| EAC | 0.66 (0.38-1.15) | 0.92 (0.48-1.75) | 0.452 | 1.02 (0.92-1.14) | 0.98 (0.84-1.14) | 0.629 | 1.13 (0.99-1.29) | 1.02 (0.84-1.24) | 0.409 |
| ESCC | 0.45 (0.17-1.17) | 0.63 (0.39-1.02) | 0.527 | 0.91 (0.59-1.42) | 0.88 (0.58-1.32) | 0.89 | 0.81 (0.61-1.08) | 0.91 (0.62-1.35) | 0.643 |
| GC | 0.78 (0.47-1.29) | **0.66 (0.45-0.98)** | 0.626 | 1.04 (0.92-1.18) | 1.01 (0.88-1.17) | 0.801 | 0.98 (0.84-1.14) | 1.02 (0.85-1.22) | 0.747 |
| CRC | **0.6 (0.45-0.78)** | **0.77 (0.60-0.98)** | 0.185 | **1.10 (1.04-1.18)** | 0.91 (0.82-1.01) | **0.001** | **1.09 (1.01-1.18)** | 0.91 (0.83-1.01) | **0.005** |
| LC | **0.56 (0.37-0.84)** | 0.75 (0.55-1.03) | 0.259 | **1.18 (1.08-1.29)** | 1.02 (0.91-1.14) | **0.037** | **1.19 (1.05-1.35)** | 1.05 (0.92-1.21) | 0.191 |
| PC | **0.57 (0.43-0.76)** | **0.7 (0.57-0.87)** | 0.252 | 1.03 (0.95-1.10) | 1.02 (0.93-1.13) | 0.97 | 1.05 (0.98-1.14) | 1.07 (0.96-1.2) | 0.845 |
| GI | **0.59 (0.50-0.68)** | **0.74 (0.65-0.85)** | **0.024** | **1.07 (1.03-1.11)** | 0.98 (0.93-1.03) | **0.007** | **1.07 (1.03-1.12)** | 1.00 (0.95-1.06) | 0.068 |

GI: gastrointestinal cancer; EC: esophageal cancer; EAC: esophageal adenocarcinoma; ESCC: esophageal squamous cell carcinoma; GC: gastric cancer; CRC: colorectal cancer; LC: liver cancer; PC: pancreatic cancer; CI: confidence interval; SD: standard deviation; sHR: subdistribution hazard ratio. Fully adjusted Fine–Gray models included the Model 2 covariates specified in the main manuscript, excluding the stratification variable. CRP: C-reactive protein.

| Table S18. Associations of per-SD increases in eGDR, TG/HDL-C, and TyG with GI cancer mortality stratified by smoking status. | | | | | | | | | |
| --- | --- | --- | --- | --- | --- | --- | --- | --- | --- |
| **Types** | **eGDR** | | | **TG/HDL-C** | | | **TyG** | | |
|  | **Never** | **Previous/current** | ***P* for interaction** | **Never** | **Previous/current** | ***P* for interaction** | **Never** | **Previous/current** | ***P* for interaction** |
|  | **sHR (95% CI)** | **sHR (95% CI)** |  | **sHR (95% CI)** | **sHR (95% CI)** |  | **sHR (95% CI)** | **sHR (95% CI)** |  |
| EC | 0.78 (0.41-1.47) | **0.61 (0.51-0.73)** | 0.477 | 1.04 (0.89-1.23) | 0.97 (0.90-1.05) | 0.433 | 1.02 (0.84-1.23) | 1.03 (0.95-1.12) | 0.897 |
| EAC | 0.86 (0.33-2.28) | 0.70 (0.47-1.03) | 0.693 | 0.95 (0.73-1.23) | 1.03 (0.94-1.13) | 0.55 | 1.01 (0.74-1.36) | 1.12 (1.00-1.26) | 0.506 |
| ESCC | 1.10 (0.22-5.45) | **0.56 (0.44-0.72)** | 0.42 | 0.71 (0.36-1.39) | 0.95 (0.68-1.32) | 0.451 | 0.80 (0.51-1.26) | 0.80 (0.51-1.26) | 0.745 |
| GC | 0.77 (0.38-1.56) | **0.69 (0.5-0.95)** | 0.771 | 0.88 (0.74-1.05) | 1.08 (0.98-1.20) | 0.05 | 0.94 (0.79-1.12) | 1.02 (0.88-1.19) | 0.48 |
| CRC | **0.53 (0.38-0.74)** | **0.70 (0.57-0.86)** | 0.158 | 1.03 (0.93-1.14) | 1.04 (0.98-1.11) | 0.846 | 1.00 (0.90-1.11) | 1.05 (0.98-1.13) | 0.453 |
| LC | 0.68 (0.37-1.25) | **0.64 (0.52-0.78)** | 0.824 | **1.25 (1.08-1.45)** | 1.08 (0.99-1.17) | 0.076 | **1.24 (1.03-1.48)** | 1.11 (0.99-1.23) | 0.287 |
| PC | **0.68 (0.47-0.98)** | **0.64 (0.54-0.76)** | 0.771 | 1.02 (0.91-1.13) | 1.02 (0.96-1.10) | 0.884 | 1.01 (0.90-1.13) | **1.08 (1.00-1.16)** | 0.352 |
| GI | **0.67 (0.54-0.82)** | **0.65 (0.58-0.73)** | 0.792 | 1.04 (0.98-1.11) | **1.04 (1.00-1.07)** | 0.841 | **1.03 (0.97-1.10)** | **1.06 (1.02-1.10)** | 0.455 |

GI: gastrointestinal cancer; EC: esophageal cancer; EAC: esophageal adenocarcinoma; ESCC: esophageal squamous cell carcinoma; GC: gastric cancer; CRC: colorectal cancer; LC: liver cancer; PC: pancreatic cancer; CI: confidence interval; SD: standard deviation; sHR: subdistribution hazard ratio. Fully adjusted Fine–Gray models included the Model 2 covariates specified in the main manuscript, excluding the stratification variable. Smoking was categorized as never versus previous/current.

| Table S19. Associations of per-SD increases in eGDR, TG/HDL-C, and TyG with GI cancer mortality stratified by alcohol consumption. | | | | | | | | | |
| --- | --- | --- | --- | --- | --- | --- | --- | --- | --- |
| **Types** | **eGDR** | | | **TG/HDL-C** | | | **TyG** | | |
|  | **Never** | **Any intake** | ***P* for interaction** | **Never** | **Any intake** | ***P* for interaction** | **Never** | **Any intake** | ***P* for interaction** |
|  | **sHR (95% CI)** | **sHR (95% CI)** |  | **sHR (95% CI)** | **sHR (95% CI)** |  | **sHR (95% CI)** | **sHR (95% CI)** |  |
| EC | **0.50 (0.32-0.79)** | **0.67 (0.53-0.84)** | 0.273 | 0.97 (0.85-1.10) | 1.00 (0.91-1.08) | 0.701 | 1.01 (0.88-1.17) | 1.03 (0.94-1.13) | 0.823 |
| EAC | **0.44 (0.21-0.91)** | 0.92 (0.56-1.52) | 0.103 | 0.95 (0.81-1.11) | 1.05 (0.95-1.15) | 0.291 | 1.07 (0.85-1.34) | 1.12 (0.98-1.26) | 0.736 |
| ESCC | 0.45 (0.18-1.13) | **0.59 (0.41-0.84)** | 0.6 | 0.80 (0.52-1.24) | 0.94 (0.65-1.35) | 0.595 | 0.88 (0.61-1.28) | 0.83 (0.61-1.13) | 0.821 |
| GC | 0.94 (0.52-1.72) | **0.61 (0.46-0.82)** | 0.206 | 1.04 (0.88-1.23) | 1.03 (0.93-1.16) | 0.979 | 0.93 (0.77-1.13) | 1.04 (0.89-1.21) | 0.4 |
| CRC | **0.52 (0.37-0.74)** | **0.71 (0.58-0.87)** | 0.147 | 1.01 (0.92-1.11) | 1.05 (0.98-1.12) | 0.505 | 1.05 (0.95-1.16) | 1.02 (0.95-1.10) | 0.699 |
| LC | 0.67 (0.42-1.07) | **0.62 (0.50-0.75)** | 0.752 | **1.14 (1.02-1.27)** | **1.11 (1.01-1.22)** | 0.719 | 1.10 (0.94-1.29) | **1.17 (1.04-1.31)** | 0.532 |
| PC | 0.79 (0.55-1.13) | **0.61 (0.52-0.72)** | 0.204 | 1.10 (1.00-1.20) | 0.98 (0.91-1.05) | 0.049 | **1.13 (1.01-1.26)** | 1.03 (0.95-1.11) | 0.153 |
| GI | **0.65 (0.54-0.78)** | **0.65 (0.57-0.73)** | 0.943 | **1.06 (1.01-1.11)** | 1.03 (0.99-1.07) | 0.289 | **1.07 (1.01-1.13)** | **1.04 (1.00-1.09)** | 0.551 |

GI: gastrointestinal cancer; EC: esophageal cancer; EAC: esophageal adenocarcinoma; ESCC: esophageal squamous cell carcinoma; GC: gastric cancer; CRC: colorectal cancer; LC: liver cancer; PC: pancreatic cancer; CI: confidence interval; SD: standard deviation; sHR: subdistribution hazard ratio. Fully adjusted Fine–Gray models included the Model 2 covariates specified in the main manuscript, excluding the stratification variable. Alcohol consumption was categorized as never versus any intake.

| Table S20. Associations of per-SD increases in eGDR, TG/HDL-C, and TyG with GI cancer mortality stratified by prediabetes status. | | | | | | | | | |
| --- | --- | --- | --- | --- | --- | --- | --- | --- | --- |
| **Types** | **eGDR** | | | **TG/HDL-C** | | | **TyG** | | |
|  | **No prediabetes** | **Prediabetes** | ***P* for interaction** | **No prediabetes** | **Prediabetes** | ***P* for interaction** | **No prediabetes** | **Prediabetes** | ***P* for interaction** |
|  | **sHR (95% CI)** | **sHR (95% CI)** |  | **sHR (95% CI)** | **sHR (95% CI)** |  | **sHR (95% CI)** | **sHR (95% CI)** |  |
| EC | **0.72 (0.57-0.92)** | **0.54 (0.33-0.88)** | 0.235 | 0.95 (0.87-1.04) | 1.01 (0.9-1.13) | 0.382 | 0.99 (0.91-1.09) | 1.06 (0.92-1.23) | 0.48 |
| EAC | 0.81 (0.51-1.28) | 0.68 (0.3-1.52) | 0.717 | 0.96 (0.85-1.07) | 1.08 (0.94-1.24) | 0.184 | 1.05 (0.92-1.19) | 1.18 (0.96-1.46) | 0.365 |
| ESCC | **0.7 (0.49-0.99)** | **0.41 (0.2-0.86)** | 0.23 | 0.76 (0.53-1.09) | 1.15 (0.79-1.68) | 0.187 | 0.78 (0.6-1.01) | 1.02 (0.64-1.62) | 0.39 |
| GC | **0.66 (0.49-0.89)** | 0.79 (0.39-1.62) | 0.648 | 1 (0.88-1.14) | 1.09 (0.94-1.25) | 0.474 | 0.97 (0.84-1.13) | 1.07 (0.86-1.32) | 0.525 |
| CRC | **0.64 (0.55-0.75)** | 0.83 (0.55-1.26) | 0.204 | 1.04 (0.97-1.12) | 1.03 (0.94-1.13) | 0.928 | 1.03 (0.95-1.1) | 1.04 (0.93-1.17) | 0.842 |
| LC | **0.64 (0.52-0.79)** | **0.46 (0.29-0.74)** | 0.322 | **1.11 (1.01-1.21)** | **1.16 (1.02-1.31)** | 0.691 | **1.14 (1.01-1.28)** | 1.18 (0.99-1.41) | 0.777 |
| PC | **0.62 (0.53-0.74)** | 0.71 (0.48-1.04) | 0.572 | 1.05 (0.98-1.12) | 0.96 (0.86-1.06) | 0.154 | 1.08 (1-1.17) | 1.01 (0.9-1.13) | 0.281 |
| GI | **0.65 (0.58-0.73)** | **0.68 (0.55-0.84)** | 0.681 | 1.03 (0.99-1.07) | 1.04 (0.99-1.09) | 0.788 | **1.04 (1-1.09)** | 1.06 (1-1.13) | 0.797 |

GI: gastrointestinal cancer; EC: esophageal cancer; EAC: esophageal adenocarcinoma; ESCC: esophageal squamous cell carcinoma; GC: gastric cancer; CRC: colorectal cancer; LC: liver cancer; PC: pancreatic cancer; CI: confidence interval; SD: standard deviation; sHR: subdistribution hazard ratio. Fully adjusted Fine–Gray models included the Model 2 covariates specified in the main manuscript, excluding the stratification variable.

| Table S21. Associations of per-SD increases in eGDR, TG/HDL-C, and TyG with GI cancer mortality stratified by lipid-lowering medication use. | | | | | | | | | |
| --- | --- | --- | --- | --- | --- | --- | --- | --- | --- |
| **Types** | **eGDR** | | | **TG/HDL-C** | | | **TyG** | | |
|  | **No** | **Yes** | ***P* for interaction** | **No** | **Yes** | ***P* for interaction** | **No** | **Yes** | ***P* for interaction** |
|  | **sHR (95% CI)** | **sHR (95% CI)** |  | **sHR (95% CI)** | **sHR (95% CI)** |  | **sHR (95% CI)** | **sHR (95% CI)** |  |
| EC | **0.49 (0.34-0.70)** | 0.8 (0.55-1.16) | 0.062 | 1.01 (0.93-1.10) | 0.94 (0.82-1.07) | 0.323 | 1.08 (0.98-1.19) | 0.94 (0.82-1.07) | 0.09 |
| EAC | 0.67 (0.38-1.18) | 0.84 (0.45-1.55) | 0.608 | 1.07 (0.96-1.18) | 0.92 (0.79-1.07) | 0.121 | 1.13 (0.98-1.31) | 1.03 (0.87-1.22) | 0.41 |
| ESCC | **0.43 (0.21-0.92)** | 0.67 (0.40-1.12) | 0.062 | 0.93 (0.70-1.24) | 0.83 (0.40-1.73) | 0.763 | 1.00 (0.77-1.31) | **0.56 (0.34-0.93)** | **0.044** |
| GC | **0.53 (0.33-0.87)** | 1.14 (0.56-2.30) | 0.084 | 0.98 (0.87-1.10) | 1.13 (0.99-1.29) | 0.118 | 0.99 (0.86-1.14) | 1.03 (0.83-1.27) | 0.752 |
| CRC | **0.60 (0.47-0.78)** | **0.72 (0.56-0.93)** | 0.322 | 1.06 (1.00-1.14) | 0.97 (0.88-1.07) | 0.128 | 1.04 (0.96-1.11) | 1.02 (0.91-1.14) | 0.832 |
| LC | **0.59 (0.41-0.87)** | **0.70 (0.55-0.89)** | 0.475 | 1.09 (0.99-1.19) | **1.16 (1.05-1.28)** | 0.344 | 1.11 (0.99-1.25) | **1.19 (1.02-1.40)** | 0.483 |
| PC | **0.66 (0.50-0.87)** | **0.64 (0.53-0.77)** | 0.834 | 1.02 (0.94-1.10) | 1.03 (0.94-1.12) | 0.841 | 1.02 (0.95-1.11) | **1.11 (1.00-1.24)** | 0.206 |
| GI | **0.59 (0.52-0.68)** | **0.72 (0.62-0.82)** | 0.06 | **1.04 (1.00-1.08)** | 1.04 (0.99-1.09) | 0.981 | **1.05 (1.00-1.09)** | **1.06 (1.00-1.12)** | 0.699 |

GI: gastrointestinal cancer; EC: esophageal cancer; EAC: esophageal adenocarcinoma; ESCC: esophageal squamous cell carcinoma; GC: gastric cancer; CRC: colorectal cancer; LC: liver cancer; PC: pancreatic cancer; CI: confidence interval; SD: standard deviation; sHR: subdistribution hazard ratio. Fully adjusted Fine–Gray models included the Model 2 covariates specified in the main manuscript, excluding the stratification variable.

| Table S22. Association of a per-SD increase in eGDR with incident GI cancer estimated using Fine–Gray competing-risk models. | | |
| --- | --- | --- |
| **Type** | **sHR (95% CI)** | ***P*** |
| EC | 0.65 (0.55-0.77) | <0.001* |
| EAC | 0.67 (0.53-0.83) | <0.001* |
| ESCC | 0.71 (0.48-1.03) | 0.071 |
| GC | 0.76 (0.58-0.99) | 0.043* |
| CRC | 0.75 (0.68-0.84) | <0.001* |
| LC | 0.70 (0.56-0.89) | 0.003* |
| PC | 0.68 (0.57-0.80) | <0.001* |
| GI | 0.74 (0.69-0.80) | <0.001* |

GI: gastrointestinal cancer; EC: esophageal cancer; EAC: esophageal adenocarcinoma; ESCC: esophageal squamous cell carcinoma; GC: gastric cancer; CRC: colorectal cancer; LC: liver cancer; PC: pancreatic cancer; CI: confidence interval; SD: standard deviation; sHR: subdistribution hazard ratio. Fully adjusted Fine–Gray models included the Model 2 covariates specified in the main manuscript; death before incident GI cancer was treated as a competing event. *Nominal Model 2 P values that remained significant after Benjamini–Hochberg FDR correction across 8comparisons (q<0.05).

Figure S1. Cumulative incidence curves of gastrointestinal (GI) cancer mortality by TG/HDL-C quartiles. (A) Esophageal cancer (EC), (B) Esophageal adenocarcinoma (EAC), (C) Esophageal squamous cell carcinoma (ESCC), (D) Gastric cancer (GC), (E) Colorectal cancer (CRC), (F) Liver cancer (LC), (G) Pancreatic cancer (PC), and (H) Pooled GI cancer mortality. Only the cumulative incidence of the outcome of interest is shown; competing deaths were incorporated in the estimation but are not displayed.

Figure S2. Cumulative incidence curves of gastrointestinal (GI) cancer mortality by TyG quartiles. (A) Esophageal cancer (EC), (B) Esophageal adenocarcinoma (EAC), (C) Esophageal squamous cell carcinoma (ESCC), (D) Gastric cancer (GC), (E) Colorectal cancer (CRC), (F) Liver cancer (LC), (G) Pancreatic cancer (PC), and (H) Pooled GI cancer mortality. Only the cumulative incidence of the outcome of interest is shown; competing deaths were incorporated in the estimation but are not displayed.

Figure S3. Association of TG/HDL-C with gastrointestinal (GI) cancer mortality using restricted cubic splines. (A) Esophageal cancer (EC), (B) Esophageal adenocarcinoma (EAC), (C) Esophageal squamous cell carcinoma (ESCC), (D) Gastric cancer (GC), (E) Colorectal cancer (CRC), (F) Liver cancer (LC), (G) Pancreatic cancer (PC), and (H) Pooled GI cancer mortality. Curves show fully adjusted cause-specific HRs (solid lines) with 95% CIs (shaded areas); knots were placed at the 10th, 50th, and 90th percentiles.

Figure S4. Association of TyG with gastrointestinal (GI) cancer mortality using restricted cubic splines. (A) Esophageal cancer (EC), (B) Esophageal adenocarcinoma (EAC), (C) Esophageal squamous cell carcinoma (ESCC), (D) Gastric cancer (GC), (E) Colorectal cancer (CRC), (F) Liver cancer (LC), (G) Pancreatic cancer (PC), and (H) Pooled GI cancer mortality. Curves show fully adjusted cause-specific HRs (solid lines) with 95% CIs (shaded areas); knots were placed at the 10th, 50th, and 90th percentiles.
